# Terminal-instar larval systematics and biology of west European species of Ormyridae associated with insect galls (Hymenoptera, Chalcidoidea)

**DOI:** 10.3897/zookeys.644.10035

**Published:** 2017-01-10

**Authors:** Jose F. Gómez, María Hernández Nieves, Severiano F. Gayubo, Jose Luis Nieves-Aldrey

**Affiliations:** 1 Facultad de Ciencias Biológicas (UCM), Departamento de Zoología y Antropología Física, C/ José Antonio Novais 2, 28040 Madrid, Spain; 2 Área de Zoología, Facultad de Biología, Universidad de Salamanca, 37071 Salamanca, Spain; 3 Museo Nacional de Ciencias Naturales (CSIC), Departamento de Biodiversidad y Biología Evolutiva, C/ José Gutiérrez Abascal 2, 28006 Madrid, Spain

**Keywords:** Chaetotaxy, cynipid galls, cryptic species, identification keys, immature stages, mouthparts *Ormyrus*, parasitoid

## Abstract

A systematic study of the genus *Ormyrus* (Chalcidoidea, Ormyridae) was conducted based on the morphology and biology of the terminal-instar larvae of ten west European species that are parasitoids of gall wasps and gallflies of the families Cynipidae, Eurytomidae and Tephritidae. The first detailed descriptions are provided of the terminal-instar larvae of these ten species using SEM images to illustrate diagnostic characters with systematic values. A key is provided for the identification of ormyrid larvae associated with galls in Europe, which is based particularly on characters of the head, mouthparts and mandibles. Although only limited informative variation in body shape was found, the setation of the head provided several characters of potential taxonomic value. The larval biology of the ten ormyrid species inhabiting different galls is also summarised. Although *Ormyrus* larvae are usually solitary idiobiont ectoparasitoids of the host larva of various gall-inhabiting insects, evidence of secondary phytophagy was observed in some species.

## Introduction

The superfamily Chalcidoidea is the second largest superfamily of parasitoid Hymenoptera (Sharkey and Fernandez 2006, New 2012, Noyes 2016) and includes 22 different extant families (Heraty et al. 2013, Noyes 2016). Within the Chalcidoidea, Ormyridae is a small family with a worldwide distribution that is composed of approximately 140 species in 3 genera. The genera *Eubeckerella* Narendran and *Ormyrulus* Bouček are monotypic, and the third is the large genus *Ormyrus*, which includes the other species (Aguiar et al. 2013, Noyes 2016). *Ormyrus* is the only genus found in the Palaearctic region (Zerova and Seryogina 2006).

The ormyrids are chalcidoids that are morphologically well characterised by their usually bright metallic colours, coarsely crenulated sculpture of the metasoma, well-developed hind coxae, short stigmal veins and two stout and curved metatibial spurs.

The family has never been catalogued or revised worldwide. References to European and Palaearctic faunas of Ormyridae are found in Erdös (1946), Bouček (1970), Nieves-Aldrey (1984), Doganlar (1984; 1991a, b) and Askew (1994). Zerova and Seryogina (2006) revised and keyed the Palaearctic species of *Ormyrus*, and subsequently, a few new species from this zoogeographic region were recently described (Lotfalizadeh et al. 2012; Zerova and Seryogina 2014a, b; Zerova et al. 2012, 2015). Hanson (1992) studied the Nearctic fauna of *Ormyrus*, and Narendran (1999) revised the Indo-Australian fauna.

The larval ormyrids are typically solitary idiobiont ectoparasitoids of various gall-forming insects. Most Holarctic species are associated with gall wasps (Hymenoptera, Cynipidae) in temperate regions, with some species linked to gall midges and gallflies (Diptera: Cecidomyiidae and Tephritidae, respectively) (Bouček 1977, Nieves-Aldrey 1984, Askew 1994, Zerova and Seryogina 2006, Noyes 2016). The galls of eurytomids (Chalcidoidea, Eurytomidae) in Spain and also those of gall-making weevils (Coleoptera, Curculionidae) in China (Askew and Blasco-Zumeta 1998, Yao and Yang 2004) are also parasitized by one *Ormyrus* species. In tropical areas, primarily in the Afrotropical and Oriental regions, some species of Ormyridae are parasitoids of the inhabitants of fig wasp galls (Chalcidoidea, Agaonidae) on *Ficus* trees (Bouček et al. 1981, Narendran 1999, van Noort 2004, Nieves-Aldrey et al. 2007, Rasplus et al. 2011). According to Simon Van Noort (pers. comm.), *Ormyrus* species are likely parasitoids or inquilines of Eurytomidae or Epichrysomallinae (Pteromalidae) that instigate fig gall formation or modify other galls.

Twenty-nine ormyrid species have been recorded in Europe, of which only seven are relatively common and widely distributed in western Europe, whereas three species, *Ormyrus salmanticus*, *Ormyrus monegricus* and *Ormyrus cupreus*, are restricted to the Iberian Peninsula. Approximately 75% of the European species of *Ormyrus* are associated with galls of Cynipidae on herbs or oak trees, with the other species linked to gall midges (Cecidomyiidae), gallflies (Tephritidae) and eurytomid galls (Eurytomidae).

Taxonomy and classification of the Ormyridae is based almost entirely on the morphological characters of the adults, with molecular data for this family remaining virtually absent (but see Hernández Nieves 2007). Given the relative uniformity of external morphological characters of the species of *Ormyrus*, biological data such as host insect species and host plant species are essential for the characterisation and identification of the species. Moreover, cryptic species have been identified in some groups of *Ormyrus* among the species associated with oak gall wasps (Hernández Nieves 2007, Graham Stone pers. comm.). However, biological data on associated plant and insect hosts are sporadic and therefore are of limited value in taxonomic approaches with Ormyridae. Phenological data, patterns of parasitism and general biological data of Ormyridae are far from complete and remain unknown for many of the European species.

Studies on the immature stages of Ormyridae are scarce or rare in the literature. Parker (1924) and Parker and Thompson (1925) are the oldest references for Ormyridae larvae. Parker (1924) contains a drawing of an ormyrid egg spiracle, whereas in Parker and Thompson (1925), ormyrids are included in a group with the Eulophidae, Elasmidae and two genera of the family Torymidae (*Megastigmus* and *Callimomus*). The group included ectoparasitic larvae with a well-defined head, thirteen body segments, an integument without pigmentation, and a barely sclerotized body with short setae or that is glabrous and four spiracles between segments II and VI. Since these classic works, only a few further papers have been published that contain information on larval morphology of the family Ormyridae. Rivosecchi (1958) provided the first detailed description of an ormyrid species, *Ormyrus hungaricus* Erdös (= *Ormyrus orientalis* Walker), which is a parasitoid of gallflies (Tephritidae) in flower heads of Asteraceae species. Later, Sellenschlo and Wall (1984) included descriptions of immature stages of Ormyridae, and Askew and Blasco-Zumeta (1998) described the larvae of *Ormyrus cupreus*. More recently, Vardal et al. (2016) provided the first data on the ovarian eggs for the family Ormyridae. However, no general study addresses the comparative morphology of the terminal larvae of *Ormyrus* species.

This study is the first comprehensive analysis of the larval morphology of the more common species of *Ormyrus* of Europe. The work is part of a wider study examining the larval morphology, biology and phylogeny of Chalcidoidea associated with gall wasps in Spain and western Europe. Studies of the larval morphology of Torymidae, Eurytomidae and Pteromalidae are completed (Gómez et al. 2008, 2011, 2013; Nieves-Aldrey et al. 2008; Gómez and Nieves-Aldrey 2012), and studies of the larval morphology of Eupelmidae and Eulophidae (Gómez and Nieves-Aldrey 2017). In this paper, we describe the terminal larvae and the biology of ten European species of Ormyridae: *Ormyrus capsalis* Askew, *Ormyrus cupreus* Askew, *Ormyrus diffinis* (Fonscolombe), *Ormyrus gratiosus* (Förster), *Ormyrus nitidulus* (Fabricius), *Ormyrus orientalis* Walker, *Ormyrus papaveris* Perris, *Ormyrus pomaceus* (Geoffroy), *Ormyrus rufimanus* Mayr and *Ormyrus wachtli* Mayr. These species are associated with insect galls on the herbaceous plants, shrubs and trees of several plant families. In this study, we aimed to contribute to the knowledge of immature stages of European ormyrid wasps in two ways. First, we identified and described larval and biological characters that are potentially useful in systematic and phylogenetic morphological work on the family Ormyridae. We used scanning electron microscopy (SEM) focused on the head capsule, mouthparts and mandibles. Second, we developed a key for the identification of terminal larvae of *Ormyrus* species associated with galls in western Europe.

## Materials and methods

**Selected taxa and specimens.** A total of 135 larval specimens belonging to ten species of Ormyridae was examined. Host galls were collected from a range of plants at sites in Spain. Sampling data are presented in Table 1. Larvae were dissected out of the galls developing on plants from different families (Asteraceae, Fagaceae, Gnetaceae, Lamiaceae, Papaveraceae and Rosaceae). Host species were identified using keys for the Iberian Cynipidae in Nieves-Aldrey (2001) and other specific key references for non-cynipid galls (Askew 1994, Askew and Blasco-Zumeta 1998). The identification of host plants species was based on Flora Europaea (Tutin et al. 1980).

**Table 1.** Summary of the host gall, host plant and sample site data for the ormyrid species included in the study. Chalcid outgroups accounted are also annotated. Depository: JLNA, JFGS and MHN; J. L. Nieves-Aldrey collection, Museo Nacional de Ciencias Naturales, Madrid, Spain.

Species	Specimens (n)	Host	Plant species	Collection data
1. *Ormyrus capsalis*	22	*Aylax minor* (Cynipidae)	*Papaver* spp. (Papaveraceae)	Spain: Monte Pajares, Rivas-Vaciamadrid, Valdemorillo (Madrid); Cabezón-San Martín de Valveni (Valladolid) (JLNA)
2. *Ormyrus cupreus*	1	*Eurytoma gallephedrae* (Eurytomidae)	*Ephedra nebrodensis* (Gnetaceae)	Spain: Monte Pajares (Madrid) (JLNA)
3. *Ormyrus diffinis*	36	*Liposthenes kerneri* (Cynipidae)	*Nepeta hispanica* (Lamiaceae)	Spain: Casa Eulogio, Rivas Vaciamadrid (Madrid) (JLNA)
4. *Ormyrus gratiosus*	11	*Isocolus scabiosae* (Cynipidae)	*Centaurea scabiosa* (Asteraceae)	Spain: Pozo de Guadalajara (Guadalajara) (JLNA)
5. *Ormyrus nitidulus*	2	*Andricus hispanicus* (Cynipidae)	*Quercus pyrenaica* (Fagaceae)	Spain: Algatocín (Málaga); Laguna de San Marcos (Salamanca) (JLNA)
6. *Ormyrus orientalis*	1	Unidentified Tephritidae (Diptera)	*Microlonchus salmanticus* (Asteraceae)	Spain: La Flecha (Salamanca) (JLNA)
7. *Ormyrus papaveris*	8	*Aylax papaveris* (Cynipidae)	*Papaver rhoeas*/*dubium* (Papaveraceae)	Spain: El Cardoso de la Sierra (Guadalajara); Rivas Vaciamadrid (Madrid) (JLNA); San Andrés (Soria) (JFG/JLNA)
8. *Ormyrus pomaceus*	1 1 1 11	*Andricus grossulariae* asex. (Cynipidae) *Trigonaspis mendesi* (Cynipidae) *Plagiotrochus fusifex* (Cynipidae) *Plagiotrochus razeti* (Cynipidae)	*Quercus faginea* (Fagaceae) *Quercus faginea* (Fagaceae) *Quercus coccifera* (Fagaceae) *Quercus ilex* (Fagaceae)	Spain: La Suara (Cádiz) (JLNA) Spain: Boadilla del Monte (Madrid) (JLNA) Spain: Arganda (Madrid) (JLNA) Spain: Villanueva del Pardillo (Madrid) (JLNA)
9. *Ormyrus rufimanus*	41	*Xestophanes potentillae* (Cynipidae)	*Potentilla reptans* (Rosaceae)	Spain: Cotos de Monterrey, Villalvilla, Villar del Olmo (Madrid); Colldejou (Tarragona) (JLNA)
10 *Ormyrus wachtli*	1	*Neaylax verbenacus*	*Salvia verbenaca* (Lamiaceae)	Spain: Arganda (Madrid) (JLNA)
EUPELMIDAE				
11. *Eupelmus cerris*	1	*Synophrus politus* (Cynipidae)	*Quercus suber* (Fagaceae)	Spain: El Pardo (Madrid) (JLNA)
EURYTOMIDAE				
12. *Eurytoma aspila*	1	*Timaspis urospermi* (Cynipidae)	*Urospermum picroides* (Asteraceae)	Spain: Algatocín (Málaga) (JLNA)
PTEROMALIDAE				
13. *Cecidostiba geganius*	1	*Andricus quercusradicis* asex. (Cynipidae)	*Quercus pyrenaica* (Fagaceae)	Spain: Miraflores (Madrid (JLNA)
TORYMIDAE				
14. *Torymus nobilis*	1	*Andricus testaceipes* asex. (Cynipidae)	*Quercus pyrenaica* (Fagaceae)	Spain: Miraflores (Madrid (JLNA)

Some parasitoid species in the families Eupelmidae, Eurytomidae, Pteromalidae and Torymidae (Hym., Chalcidoidea) associated with cynipid-galls were included in the study for comparative purposes in the systematic analysis of terminal-instar larvae (Table 1; Suppl. materials 1, 2).

**Sampling and rearing.** Samples were collected in the spring and autumn during the last 15 years with more intensive sampling in 2002–2007. Some of the galls from each sample were dissected to obtain larvae, and the remaining galls were kept separately outdoors in the open in labelled bags or were stored in rearing cages to obtain adults for identification. Some larvae from freshly dissected galls were preserved in absolute ethanol, whereas the remainder were allowed to develop to adulthood in small gelatine capsules as described by Shorthouse (1972). Information on the host galls, galled food plants and collection sites for all ormyrid species in this study are listed in Table 1. Voucher specimens of all species are deposited in the entomology collections of the Museo Nacional de Ciencias Naturales, Madrid (Spain).

**Preparation for morphological studies.** Larvae were transferred directly from absolute ethanol to a SEM stub for observation using a FEI Quanta 2000^TM^ scanning electron microscope at low vacuum without prior fixation or coating, following the method described by Nieves-Aldrey et al. (2005) for Cynipoidea and Gómez et al. (2008) for torymid larvae. Four images of each species were taken: ventral view of the larva, lateral view of the larva, anterior view of the head, and close-ups of the anterior view of the mouthparts. In addition, the right/left mandible was photographed in anterior view for some species, which involved prior dissection from the larval head, separate mounting, and gold coating for normal high vacuum observation under SEM.

**Figure 1.**
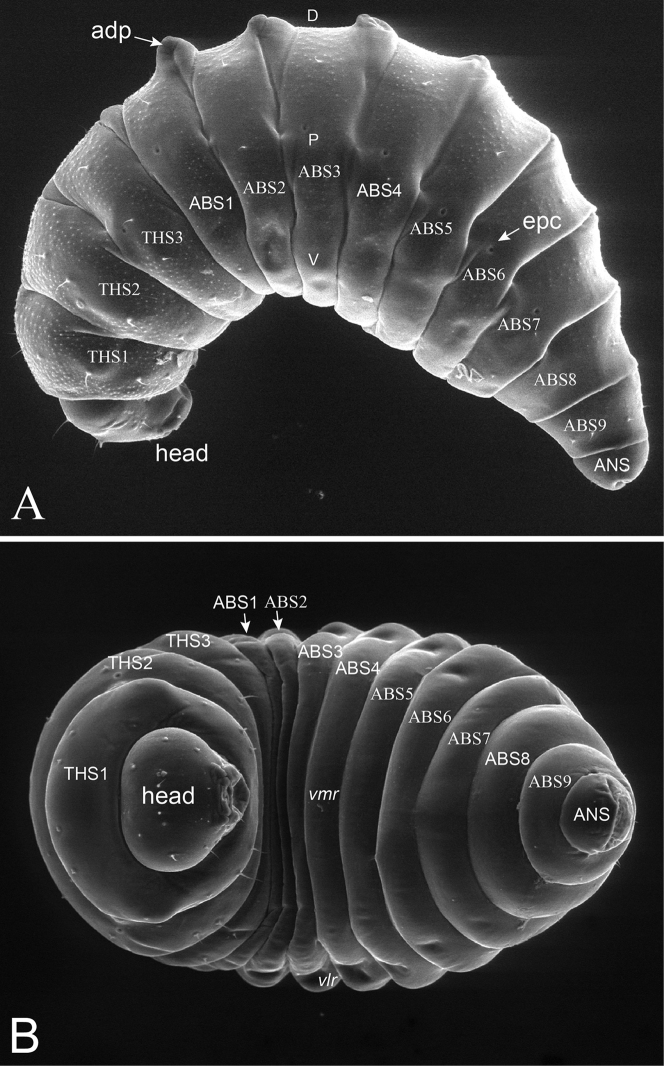
General morphology of body. **A** Lateral view of *Ormyrus cupreus* **B** ventral view of *Ormyrus diffinis*. Letters refer to the terminology used for general description (see text): *ABS1-ABS9*, abdominal segments; *adp*, anterodorsal protuberances; *ANS*, anal segment; *THS1-THS3*, thoracic segments; *D*, dorsal; *P*, pleural; *V*, ventral; *vlr*, ventrolateral region; *vmr*, ventromedial region.

**Figure 2.**
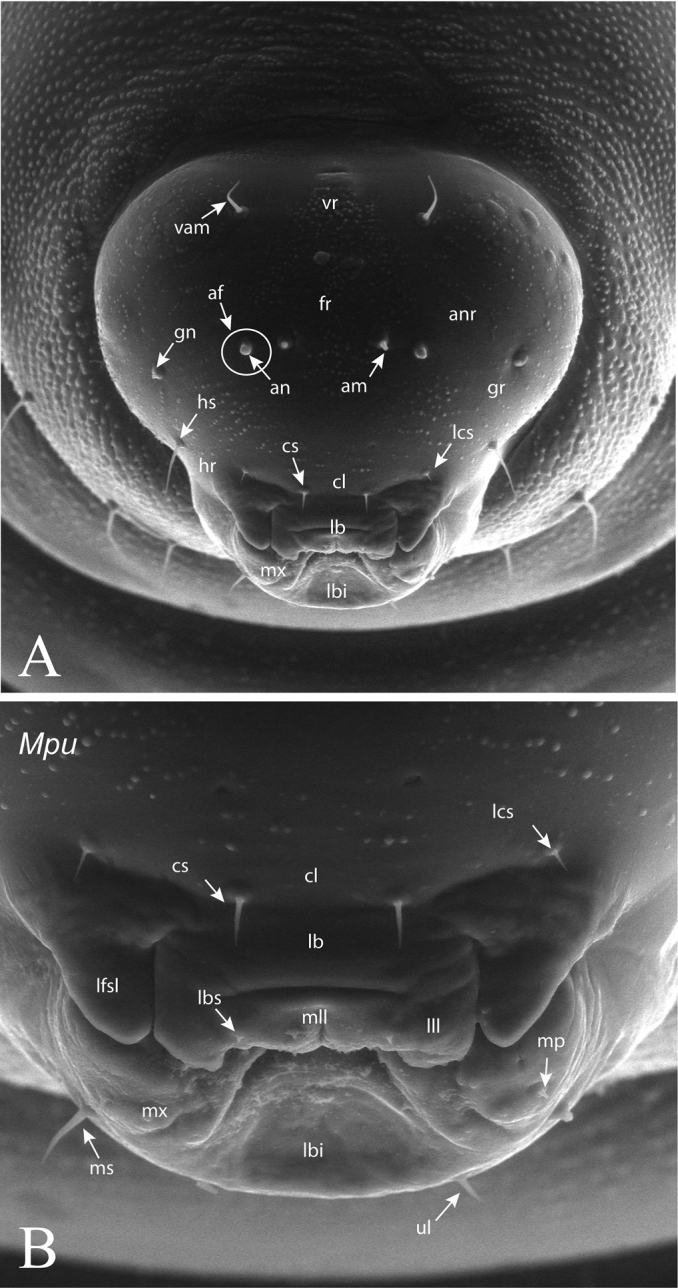
*Ormyrus nitidulus*. **A** Anterior view of head illustrating terminology used for general description (see text). Abbreviations: *af*, antennal foramina; *am*, antero-medial setae on the antennal region; *an*, antenna; *anr*, antennal area; *cl*, clypeus; *cs*, clypeal seate; *fr*, frons; *gn*, genal setae; *gr*, genal region; *hr*, hypostomal region; *hs*, hypostomal setae; *lb*, labrum; *lcs*, lateral clypeal setae; *vam*, antero-medial setae of vertex; *vr*, vertex region **B** Anterior view of mouthparts. Abbreviations: clypeus (*cl*); clypeal setae (*cs*); labrum (*lb*); lateral flaps of sides of labrum (*lfsl*); lateral lobe of labrum (*lll*); lateral clypeal setae (*lcs*); labral setae (*lbs*); medial lobe of labrum (*mll*). The under-lip complex (*Mpu*) is formed by labium (*lbi*) and maxillae (*mx*); maxillary palps (*mp*); *Mpu* setae: maxillary setae (*ms*) and antero-medial labial setae (*ul*).

**Terminology.** General terminology used in the larval descriptions follows Vance and Smith (1933) as well as Short (1952). Our terminology is also consistent with Cutler’s (1955) work on Pteromalidae larval head morphology and the referred previous studies of Gómez et al. (2008, 2011, 2013), Gómez and Nieves (2012) and Nieves-Aldrey et al. (2007) on the larvae of Torymidae, Eurytomidae and Pteromalidae. The measurements given in the descriptions were taken from samples preserved in absolute ethanol. Body length was measured as head length plus the combined length of all the remaining segments (Gómez et al. 2008). The anterodorsal protuberances (*adp*) described further below were included in the maximum body width measurement. Measurements are given as means with their range in parentheses. The ratio length/width of the body (henceforth L/W) was measured at the 3rd abdominal segment in ventral view. We also measured the ratio of the distance between antennae (SA) to the length of the antero-medial setae of the antennal area (LAA) (henceforth SA/LAA) and also to the distance between the antero-medial setae of vertex (DAV) (henceforth SA/DAV). The relative position of antennae on the head was estimated measuring the distance between the antennae to the anterior margin of clypeus related to the one between them to the upper margin of vertex (henceforth AC/AV). The ratio length/width of the first tooth of the mandible (henceforth L/W 1T) was calculated with the length of the tooth measured from the base to apex and the width measured at its base. The quantitative value of measurements is shown in Table 2. General terminology used is shown on Figures 1 and 2.

**Table 2.** Morphological measurements and ratios of studied specimens meaning as follows: body maximun length/width (L/W); head maximun length/width (HW/HL); distance between antennae/length of the antero-medial setae of the antennal area (LAA/SA); distance between antennae/distance between the antero-medial setae of vertex (DAV/SA); distance between the antennae to the anterior margin of clypeus/distance between the antennae to the upper margin of vertex (AC/AV); maximum length/width of the mandible tooth (L/W 1T).

Species	L/W	HW/HL	LAA/SA	DAV/SA	AC/AV	L/W 1T
*Ormyrus capsalis*	1.82	1.16	0.52	0.68	0.82	1.42
*Ormyrus cupreus*	1.93	1.10	0.22	1.08	1.33	–
*Ormyrus diffinis*	1.87	1.15	0.50	0.83	1.27	1.61
*Ormyrus gratiosus*	1.74	1.09	0.32	0.84	0.89	1.50
*Ormyrus nitidulus*	2.01	1.10	0.04	0.93	0.77	1.64
*Ormyrus orientalis*	1.74	0.89	0.41	0.76	1.22	–
*Ormyrus papaveris*	2.00	0.89	0.10	0.77	2.00	1.64
*Ormyrus pomaceus* ex *Plagiotrochus*	2.01	0.86	0.04	0.92	1.33	–
*Ormyrus pomaceus* ex *Trigonaspis*	1.94	0.83	0.24	0.81	1.07	1.39
*Ormyrus rufimanus*	2.01	1.00	0.20	0.93	1.10	1.80
*Ormyrus wachtli*	2.09	0.86	0.02	0.56	1.44	–

**Systematic analysis: Coding of morphological characters.** Ormyrid nomenclature followed Noyes (2016). Descriptions of the taxa were based primarily on preserved material, but with additional observations from living larvae. To standardise the comparative morphological study, the morphological variation for all the *Ormyrus* larvae was coded in an observation matrix of character states, which included coding of 28 characters related to external morphology based on SEM images. The list of characters and character states are provided in Suppl. material 1, with the subsequent matrix provided in Suppl. material 2.

## Results

### Ormyridae Förster, 1856

#### General larval morphology of *Ormyrus*

The appearance of terminal-instar larvae of *Ormyrus* is hymenopteriform (Clausen 1940) and most features are shared with other chalcidoid larvae, especially Eurytomidae, as described below. The body setae are short or almost absent on the abdominal segments, but range from short to moderately long on the thorax and head. As in eurytomids, 5–7 pairs of setae are present on the head capsule (Gómez et al. 2011, 2013). Both larvae of Eurytomidae and Ormyridae (Chalcidoidea) are superficially similar, and share the same pattern of setae over the head and body, but the mandibles clearly distinguish these two families: they are bidentate and partially visible externally in Eurytomidae, as opposed to simple single-toothed and not visible externally in Ormyridae.

The labrum of Eurytomidae and Ormyridae is also similar in being divided into a medial and two lateral lobes; however, while the medial part of the labrum of *Eurytoma* is usually divided into five lobes, the medial lobe in *Ormyrus* is usually undivided or superficially divided into three lobes.

**Body segmentation** (Fig. 1A, B). As for other hymenopteriform chalcid larvae, the body consists of the head plus 13 post-cephalic segments. Three segments form the thorax (THS1–THS3) and the remaining ten segments constitute the abdomen including the anal segment (ABS1–ABS9, ANS).

**General morphology in ventral view** (Fig. 1A). Body fusiform, relatively short and wide but slightly broader at ABS2-ABS3 level. Anal segment looks wider than long. Body integument whitish, with a pattern of short setae regularly placed in rows.

**General morphology in lateral view** (Fig. 1B). Body ventrally bent, with ventral margin of abdominal segments convex; between THS3 and ABS4 anterodorsal protuberances generally present (*adp*). Body segments divided in lateral view into three areas: pleural (P), including the spiracles (*epc*), ventral (V) and dorsal (D), over which body setae are located in three rows respectively, being abdominal setae shorter than the half of the width of an abdominal segment measured at *epc* level.

**Spiracles** (Fig. 1B). The tracheal system is composed externally of nine pairs of lateral spiracles (*epc*) opening from segment THS2 to ABS7.

**Head** (Fig. 2A). Head usually trapezoid-shaped, broader than high. Upper margin of vertex regularly rounded, with its medial area convex. Antennal area (*anr*) inconspicuous with the basal region or antennal foramina (*af*) indistinct; antennae (*an*) short but always visible on frons (*fr*), situated in the midway between clypeus (*cl*) and vertex area (*vr*). Head with 5–7 pairs of conspicuous setae always present: (i) pair of antero-medial setae on the antennal region (*am*); (ii) pair of antero-medial setae on vertex (*vam*); (iii) pair of genal setae (*gns*) on genae (*gr*); (iv) pair of clypeal setae (*cs*) on clypeus (*cl*); (v) pair of lateral clypeal setae (*lcs*) situated in lower frontal area (both iv and v with the same length); and (vi) pair of hypostomal setae. Moreover in one studied species (see *Ormyrus wachtli* later) there is a pair of extra supraclypeal setae. The clypeus (*cl*) constitutes always a more or less rectangular region with a straight ventral margin situated anterodorsally to the underlip complex (*Mpu*) and a pair of more or less extended lateral flaps on the sides of labrum (*lfsl*). The labrum (*lb*) is divided into two lateral lobes (*lll*) and a medial and undivided piece (*mll*), which is wider than lateral ones. The labrum bears a pair of labral setae (*lbs*) situated in its terminal margin.

**Mouth parts** (Figs 2A, B). Comprise the mandibles (see below) and the underlip complex (*Mpu*), which is formed by the hypopharynx (hardly discernible), the triangle-shaped maxillae (*mx*) and the labium (*lbi*). In ormyrid terminal instar larvae the labium and maxillae are clearly separated being the last discernible. The maxillary palps (*mp*) are also conspicuous and visible. Below the maxillae ventrally is the labium, usually concave and collapsed. The maxillae and labium bear two pairs of short setae, often visible: a pair of antero-medial labial setae (*ul*) and a pair of maxillary setae (*ms*) on one of the two maxillary palps.

**Mandibles** (Figs 9, 10, 11). *Ormyrus* larvae mandibles are simple, generally covered by labrum and only externally visible in part. Both are usually symmetrical and single-toothed, which is usually sharp and slightly curved on the apex.

**Taxonomy.** Descriptions of the taxa were based primarily on preserved material but with additional observations from living larvae. The diagnosis of the genus *Ormyrus* was based entirely on SEM observations and partly on previous work by Rivosecchi (1958), Sellenschlo and Wall (1984) and Askew and Blasco-Zumeta (1998). All larval descriptions and the key are new. Ormyrid nomenclature followed Noyes (2016). The key provided identifies the larvae of the ten species studied in this paper, which represented the core or most common *Ormyrus* species associated with different gall species in Europe and on the Iberian Peninsula. Some additional characters are annotated in the corresponding figures included in the key, according to the coded morphological characters listed in Suppl. material 1.

### Key to the terminal-instar larvae of the commonest *Ormyrus* species associated with European gall communities.

**Table:** 

1	Body and head integument with predominant blister-like sculpture (Figs 7B, 7:1); anteromedial setae of antennal area very short, generally < 0.3 as the distance between antennae (Fig. 7E, 15:1)	**2**
–	Body and head integument for the most part smooth; blister-like sculpture only on the genal area (Fig. 7D, 9:1); anteromedial setae of antennal area long, 0.3-0.7 the distance between antennae (Fig. 7D, 15:2)	**5**
2	Supraclypeal setae present (Fig. 8E, 17:1); anteromedial setae of antennal area situated clearly above antennae (Fig. 8E)	***Ormyrus wachtli***
–	Supraclypeal setae absent (Figs 7C, 7F); anteromedial setae of antennal area usually situated at the same level or slightly above antennae (Figs 7B, 7D; 14:0), if clearly above (Figs 7A, 7D) then supraclypeal setae absent.	**3**
3	Thoracic setae long, at least as long as the length of a thoracic segment (Figs 3B, 7B)	***Ormyrus cupreus***
–	Thoracic setae short; shorter than the length of a thoracic segment (Figs 3E, 7E)	**4**
4	Large size larvae; length reaching 3 mm (Figs 3E, 5E); blister-like sculpture mostly along head being weak on body segments (Figs 3E, 5E)	***Ormyrus nitidulus***
–	Smaller size larvae, which length rarely exceed 2 mm (Figs 4C, 6B); body segments conspicuously blister-like sculpted (Figs 4C, 6B, 8C)	***Ormyrus pomaceus***
5	Upper margin of vertex rounded continuous; convex at the medial area (Fig. 7F, 10:2)	***Ormyrus orientalis***
–	Upper margin of vertex slightly interrupted, the medial area of vertex appearing concave or depressed (Fig. 7C, 10:0)	**6**
6	Anteromedial setae of the antennal area situated at the same level or slightly above antennae (Fig. 7C); lateral lobes of labrum conspicuous and not fused with the medial piece (Fig. 9C, 22:2)	**7**
–	Anteromedial setae of the antennal area situated clearly above antennae (Fig. 7A, 14:1); lateral lobes of labrum inconspicuous, almost fused with the medial piece (Fig. 9A, 22:1)	**8**
7	Body short and wide, not abruptly tapering towards anal segment from the middle of the body (Fig. 3C); integument of thoracic segments smooth (Fig. 7C); posterior margin of medial piece of labrum convex (Fig. 9C)	***Ormyrus diffinis***
–	Body elongated and narrow, abruptly tapering towards the anal segment from the middle segments (Fig. 4D); integument of thoracic segments blister-like (Fig. 8D, 7:1); posterior margin of medial piece of labrum straight (Fig. 10B)	***Ormyrus rufimanus***
8	Body elongated and narrow, abruptly tapering towards the anal segment from the middle of the body (Fig. 4A); anteromedial setae of antennal area short, < 0.3 the distance between antennae (Fig. 8A, 15:1)	***Ormyrus papaveris***
–	Body shorter and wide, not abruptly tapering towards anal segment from the middle (Figs 3A, 3D); anteromedial setae of antennal area longer; 0.5 the distance between antennae (Fig. 7D)	**9**
9	Lateral clypeal setae situated slightly above clypeal setae; distance between lateral clypeal setae and clypeal setae, twice the distance separating clypeal setae (Fig. 9D)	***Ormyrus gratiosus***
–	Lateral clypeal setae situated at the same level of clypeal setae (Fig. 9A, 18:0); distance between lateral clypeal setae and clypeal setae, the same as the distance separating clypeal setae (Fig. 9A)	***Ormyrus capsalis***

**Figure 3.**
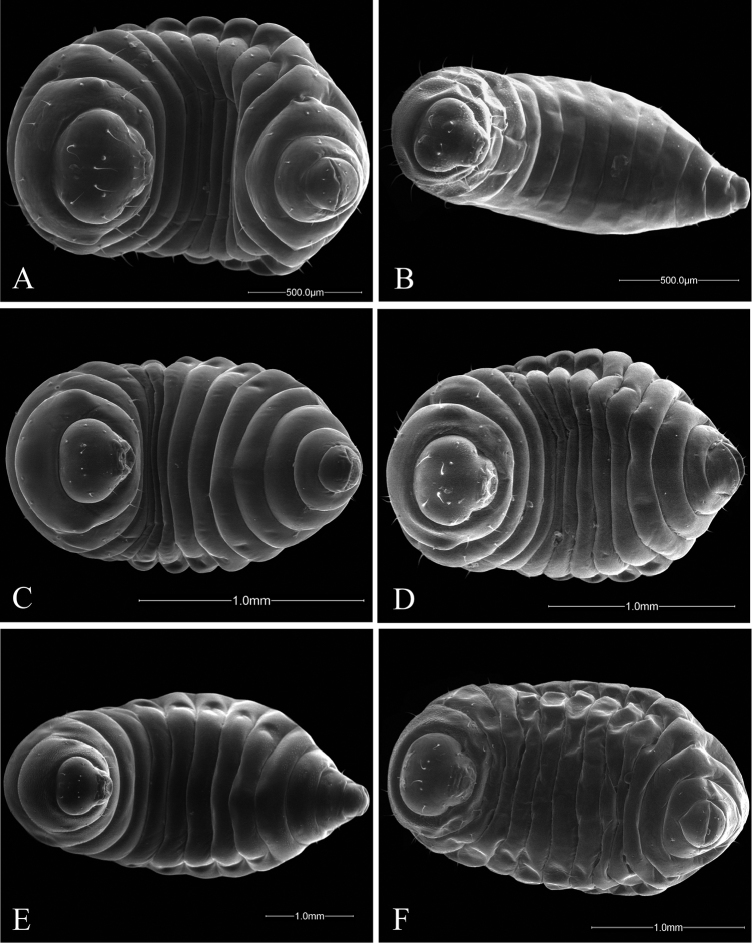
Ventral views of *Ormyrus* terminal-instar larvae. **A** *Ormyrus capsalis* **B** *Ormyrus cupreus* **C** *Ormyrus diffinis* **D** *Ormyrus gratiosus* **E** *Ormyrus nitidulus* **F** *Ormyrus orientalis*.

**Figure 4.**
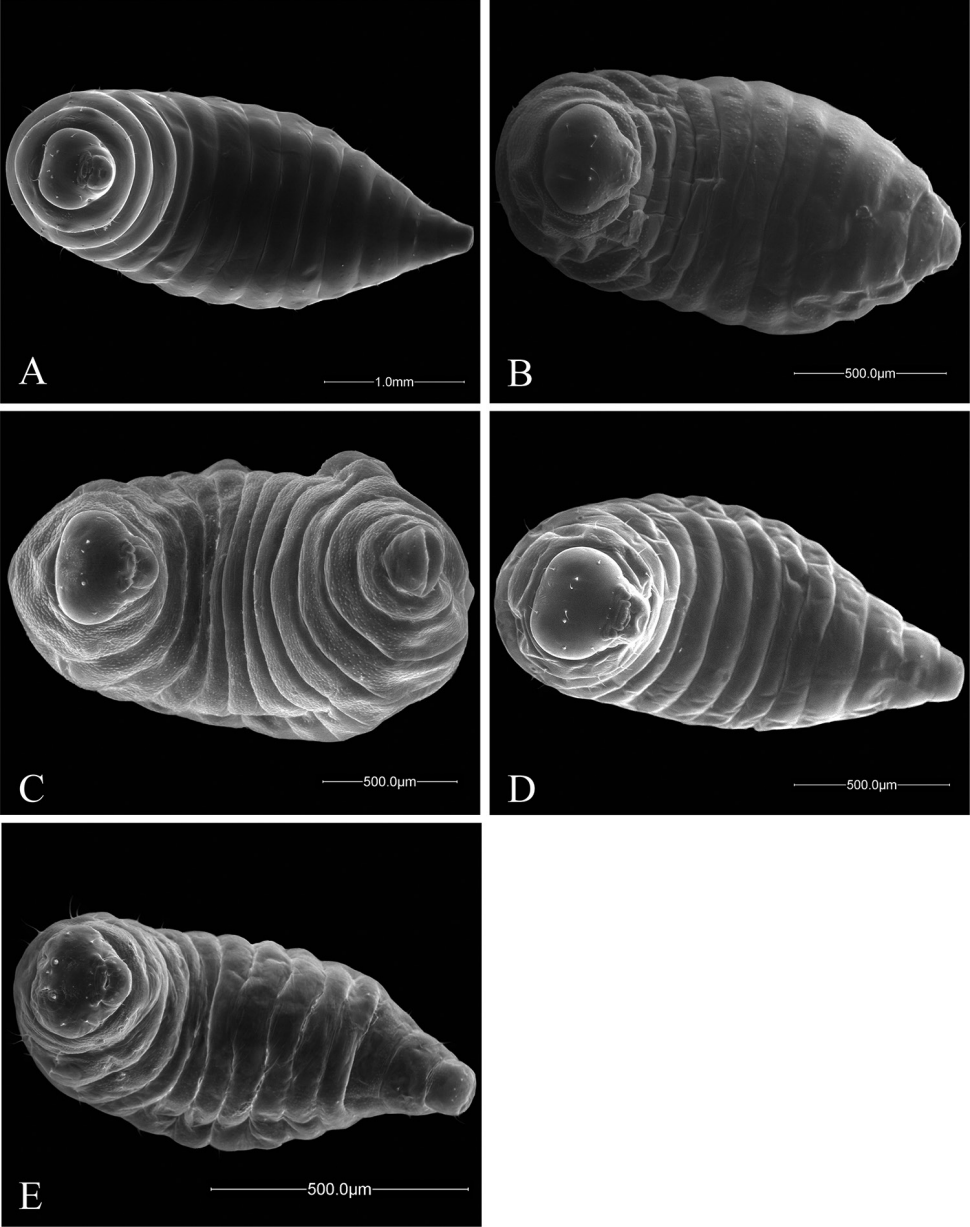
Ventral views of *Ormyrus* terminal-instar larvae. **A** *Ormyrus papaveris* **B** *Ormyrus pomaceus* ex *Trigonaspis mendesi* (Cynipidae) **C** *Ormyrus pomaceus* ex *Plagiotrochus razeti* (Cynipidae) **D** *Ormyrus rufimanus* **E** *Ormyrus wachtli*.

**Figure 5.**
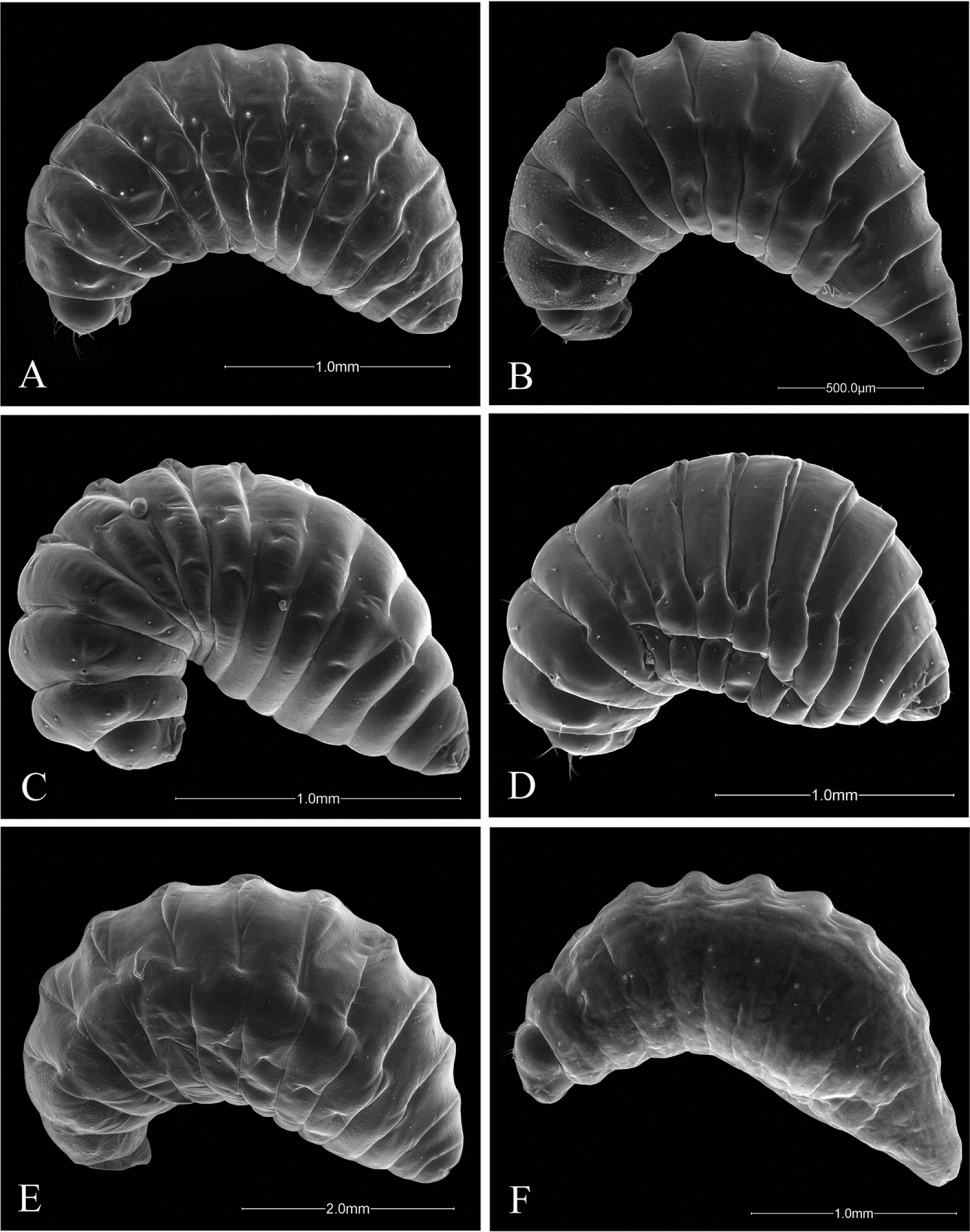
Lateral views of *Ormyrus* terminal-instar larvae. **A** *Ormyrus capsalis* **B** *Ormyrus cupreus* **C** *Ormyrus diffinis* **D** *Ormyrus gratiosus* **E** *Ormyrus nitidulus* **F** *Ormyrus papaveris*.

**Figure 6.**
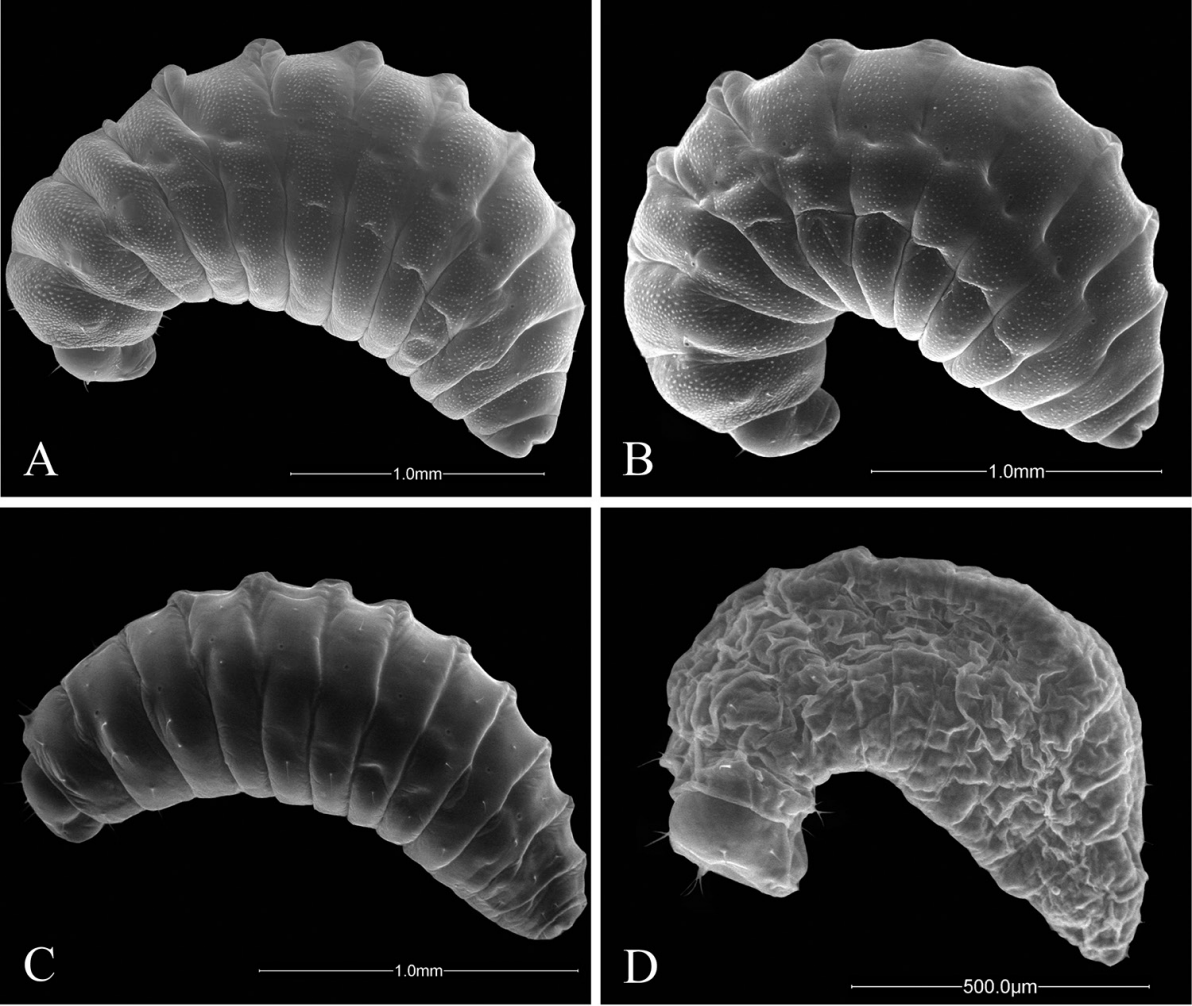
Lateral views of *Ormyrus* terminal-instar larvae. **A** *Ormyrus pomaceus* ex *Trigonaspis mendesi* (Cynipidae) **B** *Ormyrus pomaceus* ex *Plagiotrochus razeti* (Cynipidae) **C** *Ormyrus rufimanus* **D** *Ormyrus wachtli*.

**Figure 7.**
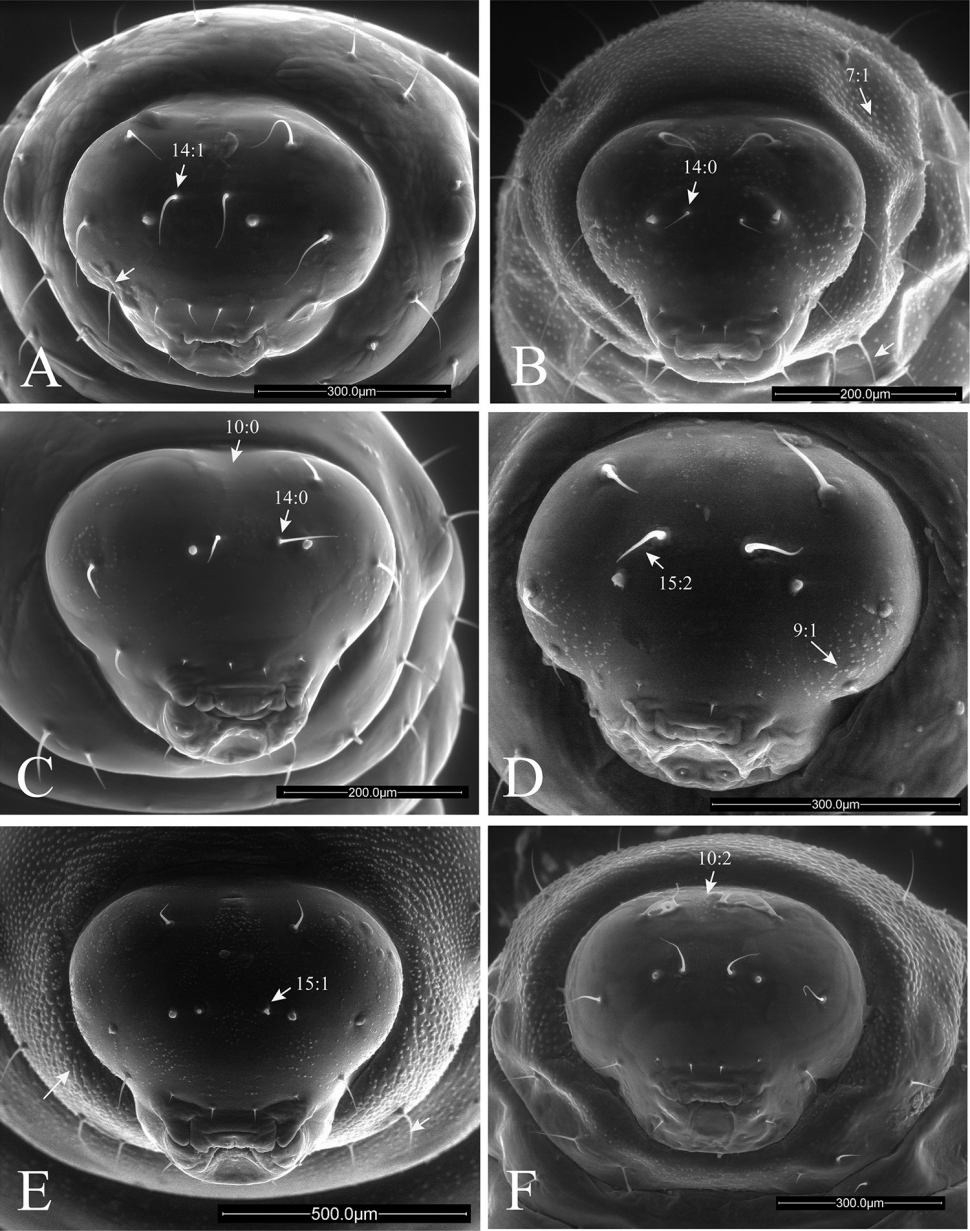
Anterior views of head of *Ormyrus* terminal-instar larvae. **A** *Ormyrus capsalis* **B** *Ormyrus cupreus* **C** *Ormyrus diffinis* **D** *Ormyrus gratiosus* **E** *Ormyrus nitidulus* **F** *Ormyrus orientalis*. Character states for outstanding features are arrowed.

**Figure 8.**
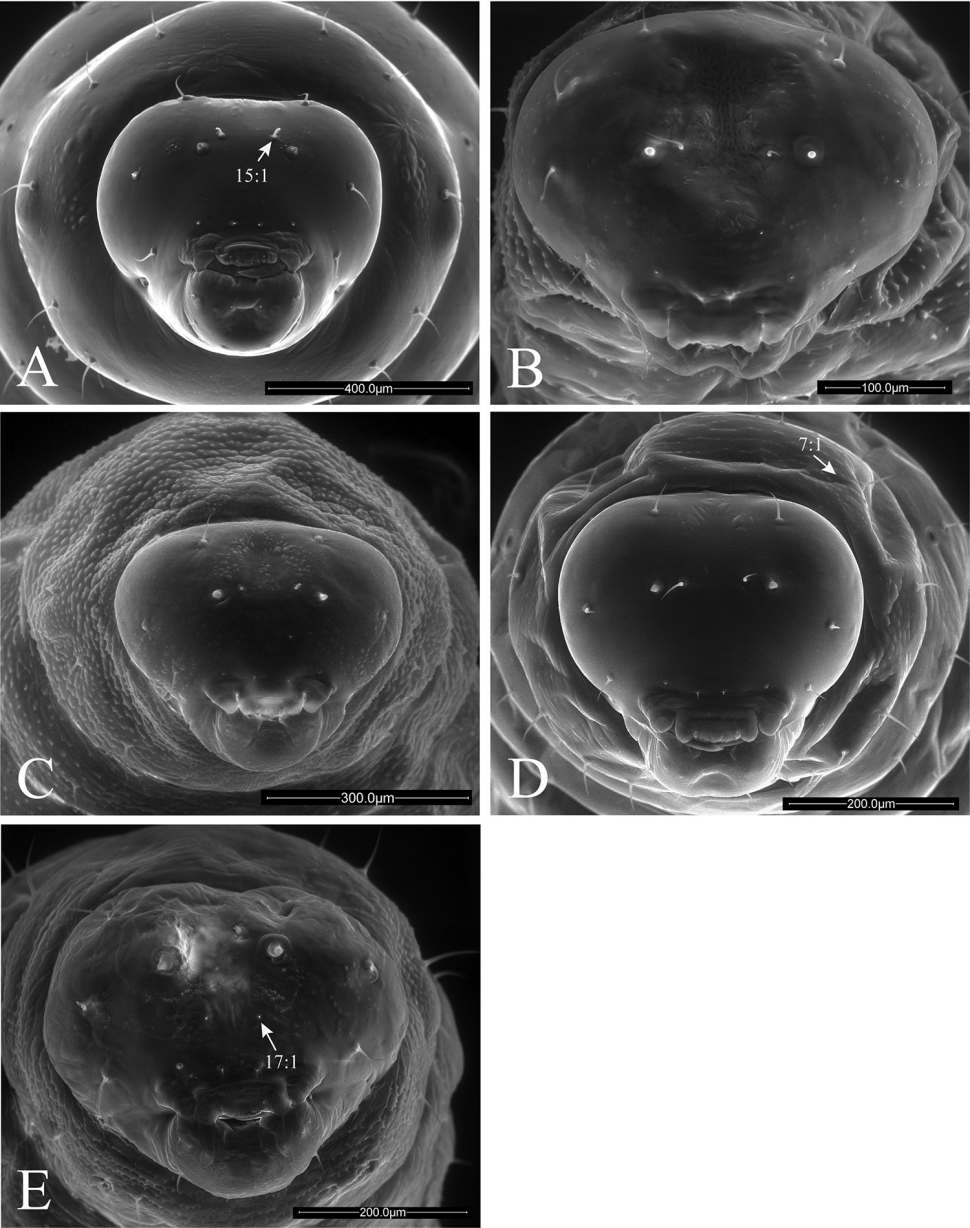
Anterior views of head of *Ormyrus* terminal-instar larvae. **A** *Ormyrus papaveris* **B** *Ormyrus pomaceus* ex *Trigonaspis mendesi* (Cynipidae) **C** *Ormyrus pomaceus* ex *Plagiotrochus razeti* (Cynipidae) **D** *Ormyrus rufimanus* **E** *Ormyrus wachtli*. Character states for outstanding features are pointed.

**Figure 9.**
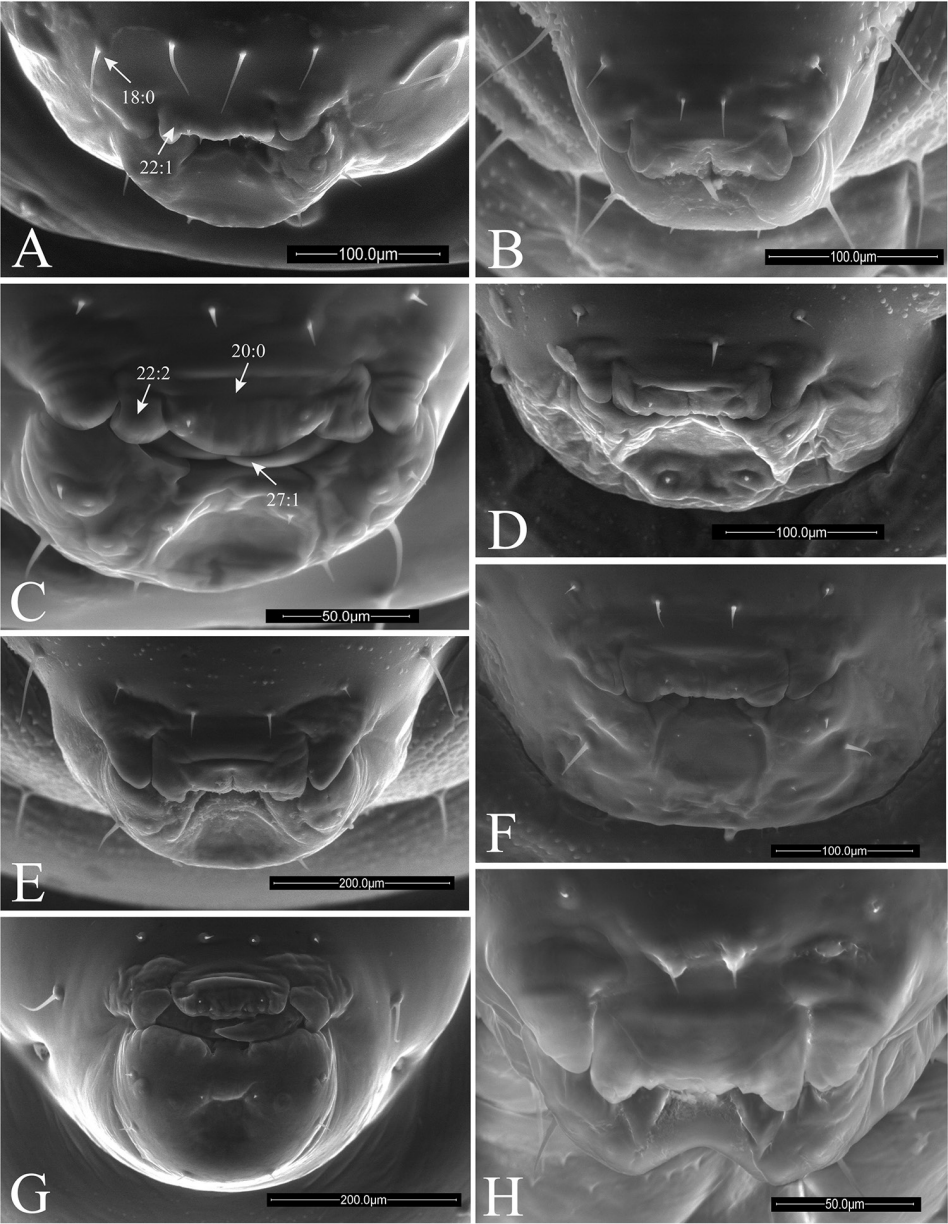
Anterior views of mouthparts of *Ormyrus* terminal-instar larvae. **A** *Ormyrus capsalis* **B** *Ormyrus cupreus* **C** *Ormyrus diffinis* **D** *Ormyrus gratiosus* **E** *Ormyrus nitidulus* **F** *Ormyrus orientalis* **G** *Ormyrus papaveris* **H** *Ormyrus pomaceus* ex *Trigonaspis mendesi* (Cynipidae). Character states for outstanding features are arrowed.

**Figure 10.**
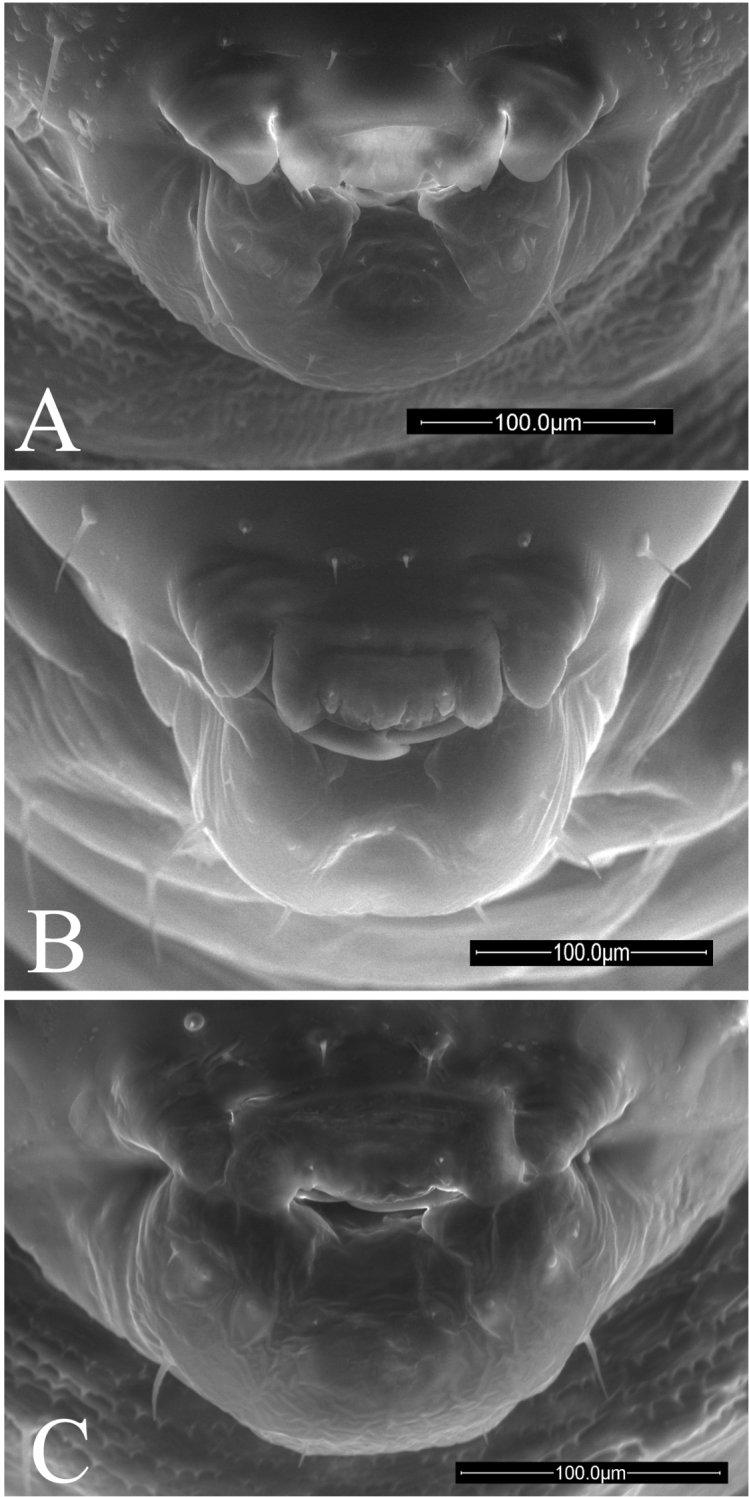
Anterior views of mouthparts of *Ormyrus* terminal-instar larvae. **A** *Ormyrus pomaceus* ex *Plagiotrochus razeti* (Cynipidae) **B** *Ormyrus rufimanus* **C** *Ormyrus wachtli*.

**Figure 11.**
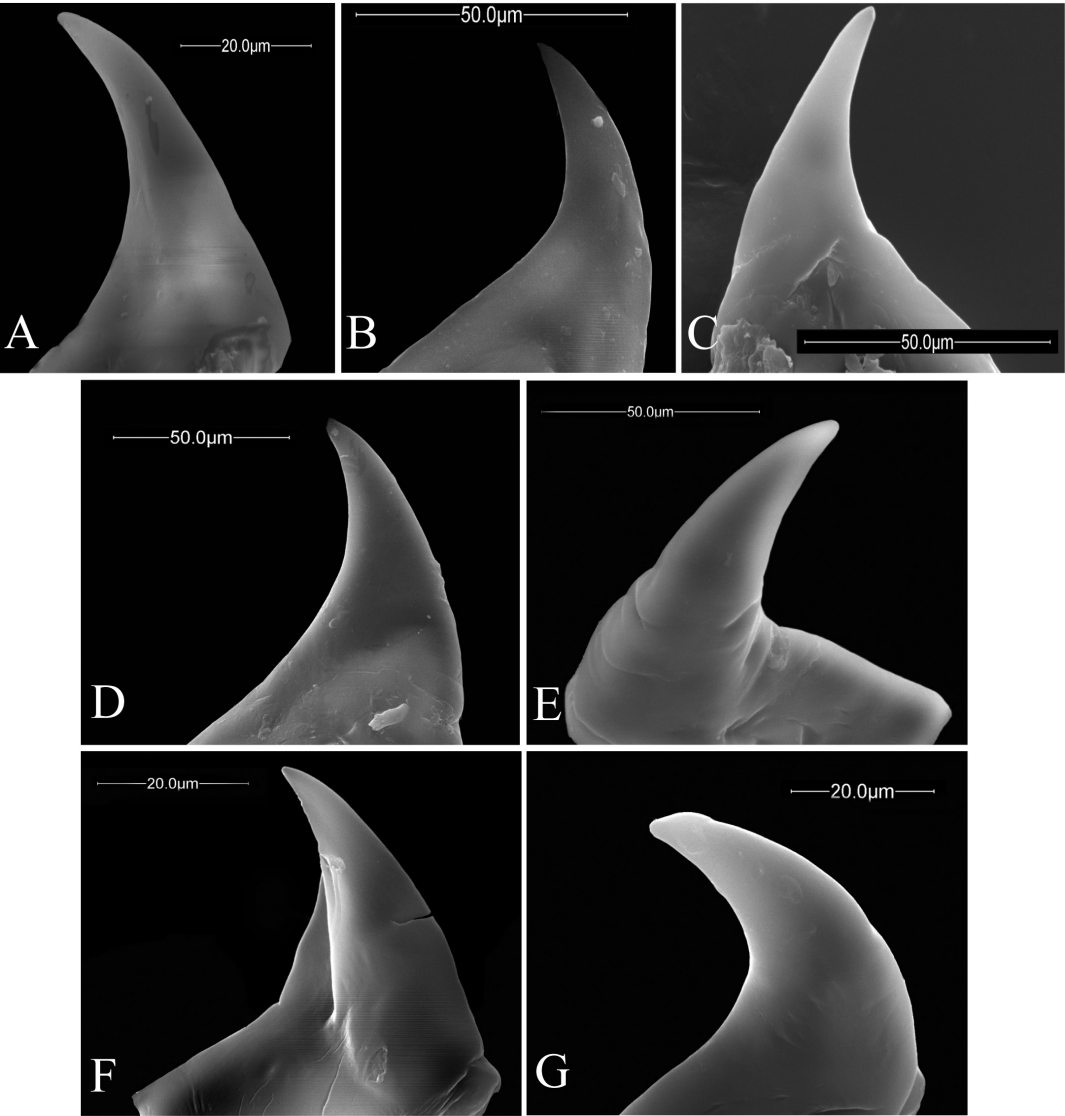
Anterior views of the right/left mandibles of *Ormyrus* terminal-instar larvae. **A** *Ormyrus capsalis* **B** *Ormyrus diffinis* **C** *Ormyrus gratiosus* **D** *Ormyrus nitidulus* **E** *Ormyrus papaveris* **F** *Ormyrus pomaceus* ex *Trigonaspis mendesi* (Cynipidae) **G** *Ormyrus rufimanus*.

**Figure 12.**
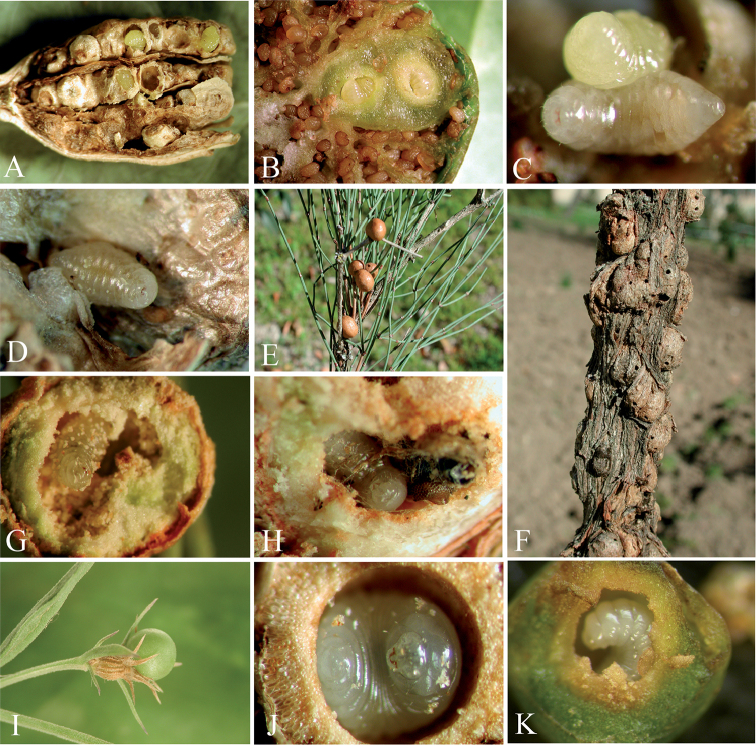
**A** Galls of *Aylax minor* on *Papaver* spp. **B** detail of cells of *Aylax minor* on *Papaver* spp. **C** larvae of *Ormyrus capsalis* on of non-parasitized larvae of *Aylax minor* **D** Detail of solitarious larvae of *Ormyrus capsalis* inside gall cell of *Aylax minor* **E** galls of *Eurytoma gallephedrae* on *Ephedra nebrodensis* **F** galls of *Eurytoma flaveola* on *Ephedra nebrodensis* **G** larvae of *Ormyrus cupreus* inside the gall cell **H** larvae of *Ormyrus cupreus* inside the gall cell with debris of adult specimen of the same species **I** gall of *Liposthenes kerneri* on *Nepeta hispanica* **J** ventral view of larvae of *Ormyrus diffinis* inside the gall cell **K** lateral view of larvae of *Ormyrus diffinis* inside the gall cell.

**Figure 13.**
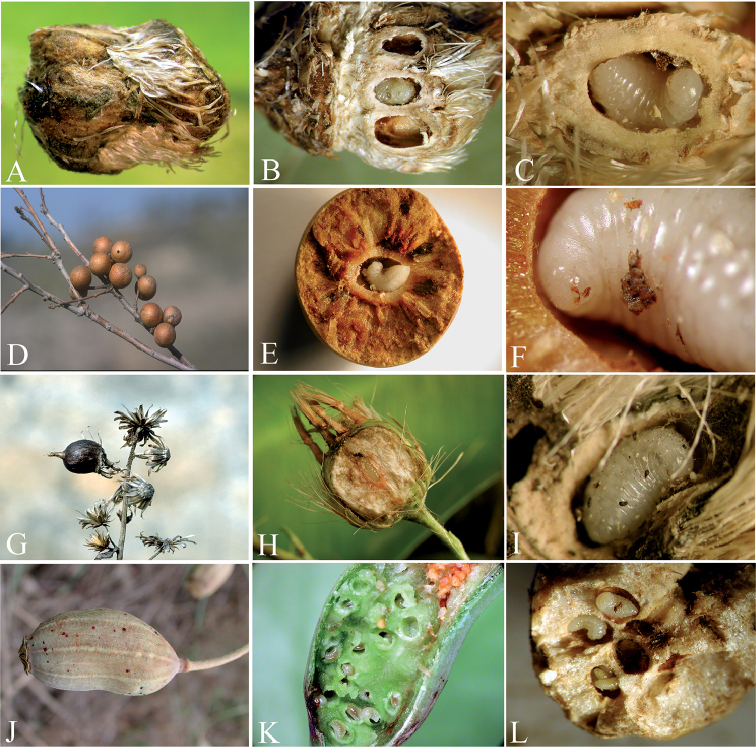
Fully (**A**) and dissected (**B**) galls of *Isocolus scabiosae* in achenes of heads of *Centaurea scabiosa*. **C** detail of larvae of *Ormyrus gratiosus* inside cells of *Isocolus scabiosae* gall **D** galls of *Andricus hispanicus* on *Quercus pyrenaica* **E** cross-section of gall of *Andricus hispanicus* with larvae of *Ormyrus nitidulus* within gall cell **F** detail of head and thorax in anterior view of larvae of *Ormyrus nitidulus* within gall cell of *Andricus hispanicus* **G** gall of *Myopites limbardae* on *Inula viscosa* **H** cross-section of gall of *Myopites limbardae* on *Inula viscosa* **I** detail of larva of *Ormyrus orientalis* within gall cell of Tephritidae on *Microlonchus salmanticus* **J** galls of *Aylax papaveris* on poppy heads **K** cross-section of poppy head shown cells of galls of *Aylax papaveris* **L** larvae of *Ormyrus papaveris* with debris of dead host within gall cells of *Aylax papaveris*.

**Figure 14.**
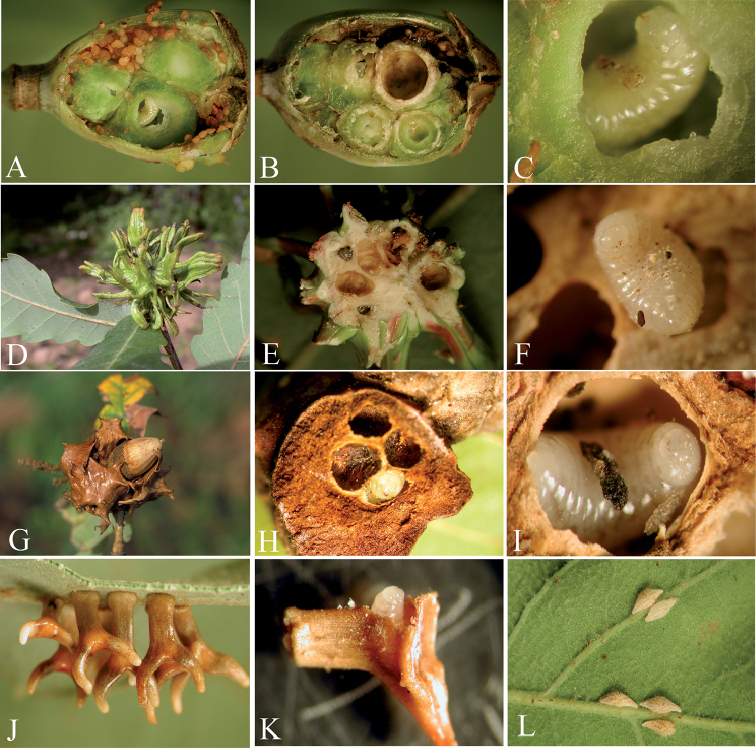
**A** cross-section of poppy head with galls of *Barbotinia oraniensis* **B** larvae of *Ormyrus papaveris* within gall of *Barbotinia oraniensis* **C** larvae of *Ormyrus papaveris* with debris of dead host within gall cells of *Barbotinia oraniensis* **D** gall of *Andricus grossulariae* (asexual) on *Quercus pyrenaica* **E** cross-section of gall *Andricus grossulariae* (asexual) **F** larvae of *Ormyrus pomaceus* ex gall of *Andricus grossulariae* **G** gall of *Andricus pictus* on *Quercus pyrenaica* **H** cross-section of gall *Andricus pictus* **I** larvae of *Ormyrus pomaceus* ex gall of *Andricus pictus* **J** galls of *Trigonaspis mendesi* on *Quercus faginea* **K** larvae of *Ormyrus pomaceus* within galls of *Trigonaspis mendesi* on *Quercus faginea* **L** galls of *Trigonaspis brunneicornis* on *Quercus faginea*.

**Figure 15.**
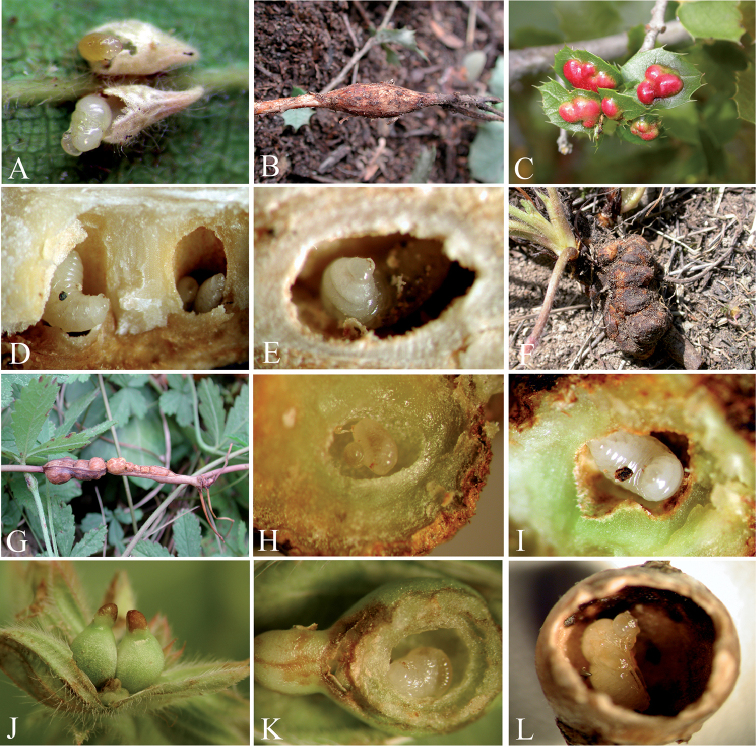
**A** larvae of *Ormyrus pomaceus* and *Trigonaspis brunneicornis* within gall cells **B** gall of *Plagiotrochus razeti* (asexual) on *Quercus* **C** galls of *Plagiotrochus quercusilicis* on *Quercus* **D** larvae of *Ormyrus pomaceus* within cells of galls of *Plagiotrochus razeti* **E** detail of larva of *Ormyrus pomaceus* within cell of gall of *Plagiotrochus razeti* **F** galls of *Xestophanes potentillae* on subterranean rhizome of *Potentilla reptans* **G** galls of *Xestophanes potentillae* on air runners of *Potentilla reptans* **H** larvae of *Ormyrus rufimanus* on larvae of *Xestophanes potentillae* within gall cell **I** larvae of *O rufimanus* with debris of dead host within gall cells of *Xestophanes potentillae*. **J** galls of *Neaylax verbenacus* on *Salvia verbenaca* **K** larvae of *Ormyrus wachtli* on larvae of *Neaylax verbenacus* within gall cell **L** pupae of *Ormyrus wachtli* within gall of *Neaylax salviae*.

### Descriptions of terminal larvae and biology of *Ormyrus* species

#### Ormyrus capsalis

Taxon classificationAnimaliaHymenopteraOrmyridae

Askew, 1994

##### Material examined.

ex gall *Aylax minor* Hartig on *Papaver* spp., Spain, Guadalajara: Valdenoches, 31.VII.01, J. L. Nieves leg (n = 1); Madrid: Monte Pajares, 7.IX.03, J. L. Nieves leg (n = 11); Madrid: Rivas-Vaciamadrid, 14.V.03, J. L. Nieves leg (n = 1); Madrid: Valdemorillo, 13.VI.04, J. L. Nieves leg (n = 2); Valladolid: Cabezón-San Martín de Valveni, 22.VI.02, J. L. Nieves leg (n = 7).

##### Description.

n = 22; Body length: 1.68 ± 0.33 mm (min-max: 1.13-2.20 mm), width: 0.92 ± 0.16 mm (min-max: 0.67-1.20 mm). Body fusiform, relatively short and wide, slightly wider at the level of ABS2-ABS3, but not tapering abruptly towards ANS (Figs 3A, 5A) (Table 2); *adp* present from the second thoracic to fifth abdominal segment, not protruding beyond the dorsal margin of body in lateral view (Fig. 5A); integument of the body smooth; thoracic setae longer than abdominal setae but shorter than length of a thoracic segment Head 1.14 broader than long (Fig. 7A); vertex concave; distance among *vam* longer than SA; *am* situated clearly above the antennae (Table 2). On clypeus *lcs* as long as *cs* (Fig. 9A), being both situated at the same level; *lll* not clearly differentiated and almost merged with medial labrum lobe; posterior margin of labrum straight. Mandibles one-toothed with apex of tooth clearly sharp (Table 2).

##### Biology.

This species is a common parasitoid in poppy galls of *Aylax minor* Hartig, 1840 (Hym., Cynipidae) (Fig. 12A and B). The species has also been reared from galls of *Aylax papaveris* and *Barbotinia oraniensis* on the heads of *Papaver* ssp. (Askew et al. 2006). The parasitoid behaviour of *Ormyrus capsalis* is very similar to the related species *Ormyrus papaveris*, an idiobiont ectoparasitoid of cynipid larvae (Askew et al. 2006) (Fig. 12C).

Notably, in some cases, we observed terminal-instar larvae of *Ormyrus* inside cells of *Aylax minor*, which apparently were not consumed (Fig. 12D).

#### Ormyrus cupreus

Taxon classificationAnimaliaHymenopteraOrmyridae

Askew, 1998

##### Material examined.

ex gall *Eurytoma gallephedrae* Askew on *Ephedra nebrodensis*, Spain, Madrid: Monte Pajares, 24.I.04, J. L. Nieves leg (n=1).

##### Description.

n = 1; Body length: 1.5 mm, width: 0.61 mm. Body fusiform, broader at the level of abdominal segments ABS2-ABS3 and tapering posteriorly towards ANS; ANS broader than long (Figs 3B, 5B) (Table 2); *adp* present from second thoracic to fifth abdominal segment, protruding conspicuously beyond the dorsal margin of body in lateral view, but only at the level of abdominal area (Fig. 5B); thoracic and abdominal segments with blister-like sculpture; thoracic setae relatively long, as long as the length of a thoracic segment. Head 1.1 broader than high (Fig. 7B); blister-like sculpture extended over the head; vertex concave in the middle; distance among *vam* as distance SA; *am* situated clearly above the antennae (Table 2). The *lcs* situated above *cs*; *cs* separated from *lcs* 2.5 as distance between *cs* (Fig. 9B). Lateral lobes of labrum almost fused with the medial lobe; posterior margin of medial piece of medial lobe straight. Maxilary palps indistinct. Mandibles unidentated with apex of tooth acute (Table 2).

##### Biology.

The larva of *Ormyrus cupreus* was described as a specific parasitoid of galls induced by *Eurytoma gallephedrae* Askew (Chalcidoidea, Eurytomidae) on *Ephedra nebrodensis* stems (Fig. 12E). Additionally, from this host, we also reared larvae and adults of *Ormyrus cupreus* from galls on subterranean runners of *Ephedra nebrodensis* (Fig. 12F), most likely induced by *Eurytoma flaveola* (Zerova 1796), which is a species recorded inducing galls on *Ephedra* roots in Asia (Zerova 1995). Askew and Blasco-Zumeta (1998) performed detailed observations on the biology of this species and found *Ormyrus cupreus* is a primary, solitary idiobiont ectoparasitoid of the larva of *Eurytoma gallephedrae*, also attacking the adult *Eurytoma* at times or as a hyperparasitoid attacking larvae of the eupelmids *Brasema* and *Eupelmus*. These authors also reported cannibalistic behaviour. Because the remains of an adult *Eurytoma* were found jointly with a larva of *Ormyrus cupreus*, we confirmed the observations of Askew (Fig. 12G and H).

#### Ormyrus diffinis

Taxon classificationAnimaliaHymenopteraOrmyridae

(Fonscolombe, 1832)

##### Material examined.

ex gall *Liposthenes kerneri* (Wachtl) on *Nepeta hispanica* Spain, Madrid: Casa Eulogio, 01.VI.03, J. L. Nieves leg (n = 8); Madrid: Rivas-Vaciamadrid, 01.VI.03, J. L. Nieves leg (n = 5); Madrid: Rivas-Vaciamadrid, 13.VI.03, J. L. Nieves leg (n = 1); Madrid: Rivas-Vaciamadrid, 17.V.03, J. L. Nieves leg (n = 22)

##### Description.

n = 36; Body length: 1.56 ± 0.34 mm (min-max: 1.00-2.40 mm), width: 0.83 ± 0.12 mm (min-max: 0.60-1.00 mm). Body fusiform, not tapering abruptly towards anal segment (Figs 3C, 5C) (Table 2); *adp* present from second thoracic to fifth abdominal segment protruding beyond the dorsal margin of body in lateral view (Fig. 5C); integument smooth; thoracic setae relatively long, abdominal setae shorter. Head 1.12 times wider than high (Fig. 7C); integument of genal area with blister-like sculpture; *vam* more separated than distance SA; *am* situated slightly above antennae (Table 2).

On clypeus *lcs* situated at the same level of *cs*; lateral lobes of labrum strongly remarked and incompletely fused with the medial lobe; posterior margin of medial piece of labrum convex (Fig. 9C); mandible unidentate with the apex slightly visible under *lb*; tooth acute (Fig. 11B).

##### Biology.

The species is a common parasitoid reared from cynipid galls of *Liposthenes kerneri* (Wachtl) on fruits of *Nepeta* ssp. (Lamiaceae; Fig. 12I) (Askew et al. 2006). Full-growth larva of *Ormyrus diffinis* occupied the entire primary cell of a parasitized gall after the host larvae was devoured (Fig. 12J and K). We observed that larvae of *Ormyrus diffinis* inside galls apparently entered prolonged periods of diapause, without causing normal pupation and adult emergence after the winter diapause period. Moreover, live terminal-instar larvae of *Ormyrus diffinis* were found in galls dissected two years after collection. The data indicate that *Ormyrus diffinis* is an idiobiont ectoparasitoid with a univoltine life cycle that is synchronized with the emergence and growth of their host galls on species of *Nepeta*. The insects emerge in the second year when the new galls are available again on the host plant. This species has also been reared from galls of *Neaylax salviae* and *Neaylax nemorosae* on different species of *Salvia* (Lamiaceae) and from those of *Rhodus cyprius* on *Salvia triloba* (Lamiaceae) (Askew et al. 2006).

#### Ormyrus gratiosus

Taxon classificationAnimaliaHymenopteraOrmyridae

(Förster, 1860)

##### Material examined.

ex gall *Isocolus scabiosae* (Giraud) on *Centaurea scabiosa*, Spain, Guadalajara: Pozo de Guadalajara, 31.VII.02, J. L. Nieves leg (n = 4); Pozo de Guadalajara, 03.X.04, J. L. Nieves leg (n = 7).

##### Description.

n = 11; Body length: 2.22 ± 0.63 mm (min-max: 1.40-3.60 mm), width: 1.28 ± 0.31 mm (min-max: 0.67-1.87 mm). The species differs from *Ormyrus capsalis* in the following characters: head 1.1 times as wide as high; genal area, vertex and first thoracic segment with blister-like sculpture; antennae mid-situated in anterior view of the head; antennal setae 0.35 as long as distance between antennae; *lcs* situated above *lc* (Figs 3D, 5D, 7D; Table 2).

##### Biology.

Larvae of *Ormyrus gratiosus* are oligophagous idiobiont ectoparasitoids of species of *Isocolus* that induce galls on flower heads of *Centaurea* and *Serratula* species (Asteraceae) (Askew et al. 2006). Additionally, the species has been reared from the galls of *Diastrophus mayri* on *Potentilla argentea* (Rosaceae). Our examined material was from dissected galled achenes of the flower heads of *Centaurea scabiosa* in Spain (Fig. 13A, B, and C).

#### Ormyrus nitidulus

Taxon classificationAnimaliaHymenopteraOrmyridae

(Fabricius, 1804)

##### Material examined.

ex gall *Andricus hispanicus* on *Quercus canariensis*, Spain, Málaga: Algatocín, 19.VIII.02, J. L. Nieves leg (n = 1); ex gall *Andricus hispanicus* on *Quercus faginea*, Spain, Salamanca: Laguna de San Marcos, 26.VIII.03, J. L. Nieves leg (n = 1)

##### Description.

n = 2; Body length: 4.28 ± 0.87 mm (min-max: 3.67-4.90 mm), width: 2.13 ± 0.18 mm (min-max: 2.00-2.25 mm).

The larva of this species is the largest among all the European species. Is quite similar in most diagnostic characters to the larvae of the related species *Ormyrus pomaceus*, being differentiated by its large size and the blister like sculpture much less conspicuous. Other diagnostic characters are as follows: body short and wide, not tapering towards the anal segment. Setae of thoracic segments shorter than ½ length of a thoracic segment; ratio AC/AV 0.77, the shortest among all the studied species (Table 2); anteromedial seatae of antennal area short, 0.3 as long as distance among antennae; *lcs* separated from *cs* 0.7 times the distance between *cs*; maxillary palps conspicuous (Figs 3E, 5E, 7E; Table 2).

##### Biology.

The species *Ormyrus nitidulus* is a member, with the closely allied *Ormyrus pomaceus*, of the parasitoid community associated with oak gall wasps (Hymenoptera, Cynipini). The two species were reared from more than 50 different species of cynipids associated with *Quercus* species in the west Palaearctic (Askew et al. 2013); however, *Ormyrus nitidulus* is not as common and is less polyphagous than *Ormyrus pomaceus*. In contrast to the closely related species *Ormyrus pomaceus*, *Ormyrus nitidulus* prefers to attack the large galls of asexual generations of heteroecic species of *Andricus*. On the Iberian Peninsula, *Ormyrus nitidulus* was reared primarily from galls of *Andricus hispanicus* (Fig. 13D, E, and F) and the asexual generation of *Andricus grossulariae*. Our observations of dissected galls showed the larva of *Ormyrus nitidulus* was a primary ectoparasitoid of the galling inducer. In the host galls of *Andricus hispanicus*, the larvae always occupied the host central larval chamber, not the secondary cells occupied by inquilines.

#### Ormyrus orientalis

Taxon classificationAnimaliaHymenopteraOrmyridae

Walker, 1871

##### Material examined.

ex gall of an undetermined Tephritidae (Diptera) on *Microlonchus salmanticus*, Spain, Salamanca: La Flecha (23/X/02), J. L. Nieves leg (n = 1).

##### Description.

n = 1; Body length: 2.35 mm, width: 1.35 mm

Body fusiform, short and wide, slightly wider at the level of ABS2-ABS3, but not tapering abruptly towards ANS (Fig. 3F) (Table 2); body segments with conspicuous blister-like sculpture; thoracic setae relatively long, clearly shorter than abdominal setae. Head 1.14 as wide as high (Fig. 7F); face integument smooth; medial area of vertex regularly convex; antennae situated at mid distance among vertex and ventral margin of clypeus; ratio AC/AV 1.22; *am* situated at the same level of antennae; antennal setae 0.4 as long as distance among antennae (Table 2).

On clypeus *lcs* situated at the same level of *cs*, both equal in length (Fig. 9F); lateral lobes of labrum inconspicuous and almost fused with the medial lobe; posterior margin of the medial piece of labrum straight; mandibles unidentated with the apex of tooth acute (Table 2).

##### Biology.

In contrast to most European species of *Ormyrus*, the larvae of *Ormyrus orientalis* attack dipteran galls induced by tephritids (Diptera, Tephritidae) in the heads of different species of Asteraceae. On the Iberian Peninsula, tephritid galls containing *Ormyrus orientalis* were found on *Microlonchus salmanticus* (Asteraceae) (Fig. 13I), and the species was also reared from galls of *Myopites limbardae* Schiner (Tephritidae) on *Inula viscosa* (Asteraceae) (Fig. 13G and H). Based on our unpublished data from Malaise traps and sweep net samples, *Ormyrus orientalis* was one of the most abundant ormyrid species in many habitats on the Iberian Peninsula; consequently, the list of hosts could be wider than that reported in the literature and in the data of the authors.

#### Ormyrus papaveris

Taxon classificationAnimaliaHymenopteraOrmyridae

Perris, 1840

##### Material examined.

ex gall *Aylax papaveris* on *Papaver* spp., Spain, Guadalajara: El Cardoso de la Sierra, 30.VI.02, J. L. Nieves leg. (n = 4); Soria: San Andrés, 14.VII.05, J. L. Nieves & J. F. Gómez leg. (n = 1); ex gall *Barbotinia oraniensis* on *Papaver* spp., Spain, Madrid: Rivas-Vaciamadrid, 25.V.02, J. L. Nieves leg. (n = 2); Madrid: Rivas-Vaciamadrid, 13.VI.04, J. L. Nieves leg. (n = 1).

##### Description.

n = 8; Body length: 1.88 ± 0.24 mm (min-max: 1.53-2.13 mm), width: 0.94 ± 0.19 mm (min-max: 0.67-1.20 mm). This species is similar to *Ormyrus capsalis* from which may be distinguished in the body fusiform, slightly wider at the level of body segments ABS2-ABS3, tapering towards the ANS (Figs 4A, 5F) and the anteromedial setae of antennal area being relatively short, <0.3 the distance among antennae. Other descriptive diagnostic characters as follows: thoracic setae short; head 1.07 times wider than high (Fig. 8A); face integument smooth; antennae situated at mid position in the face; *am* short and situated above antennae (Table 2). On clypeus *lcs* situated at the same level of *cs*, both equal in length but short and inconspicuous (Fig. 9G); lateral lobes of labrum conspicuous and clearly separated from the medial lobe; mandibles with a single tooth with acute apex (Fig. 11E).

##### Biology.

The larvae of *Ormyrus papaveris* are common ectoparasitoids in poppy galls of different Aylacini (Cynipidae) species, primarily *Aylax papaveris* and *Barbotinia oraniensis* (Figs 13J and K; 14A), and attack the host in the early stages of development (Askew et al. 2006). We observed the remains of the host larva on the body of a mature ormyrid larva (Fig. 14C). The host larval chamber of *Barbotinia oraniensis* was normally spherical and regular (Fig. 14B); however, when *Ormyrus papaveris* attacked the host, the chamber was shorter and irregular (Fig. 14C). The larva of *Ormyrus papaveris* moved inside the host gall larval cell touching the gall chamber walls with their mandibles, which suggested that during the terminal larval stage, *Ormyrus papaveris* might exhibit a similar phytophagous behaviour to that of *Eurytoma* species inhabiting galls (Askew and Blasco Zumeta 1998, La Salle 2005). In the galls of *Aylax papaveris*, the host larval cells were regularly ellipsoidal and were coated with a thin scum, whereas the cells attacked by ormyrid larvae were larger, more irregular and lacked the thin scum. Because we observed “in vivo” in dissected galls, the phytophagous behaviour of the *Ormyrus* larvae during their final larval stage caused the change in gall morphology.

#### Ormyrus pomaceus

Taxon classificationAnimaliaHymenopteraOrmyridae

(Geoffroy, 1785)

##### Material examined.

ex gall *Andricus grossulariae* asex. on *Quercus faginea*, Spain, Cádiz: La Suara-Jérez, 16.X.04, J. L. Nieves leg. (n = 1); ex gall *Plagiotrochus fusifex* on *Quercus coccifera*, Spain, Madrid: Arganda, 01.VI.03, J. L. Nieves leg. (n = 1); ex gall *Plagiotrochus razeti* on *Quercus ilex*, Spain, Madrid: Villanueva del Pardillo, 07/X/02, J. L. Nieves leg. (n = 11). ex gall *Trigonaspis mendesi* on *Quercus faginea*, Spain, Madrid: Boadilla del Monte, 23/IX/02, J. L. Nieves leg. (n = 1).

##### Description.

Ex gall *Andricus grossulariae* asex., on *Quercus faginea*, n = 1; Body length: 2.73 mm, width: 1.67 mm; ex gall *Plagiotrochus fusifex* on *Quercus coccifera*, n = 1; Body length: 1.13 mm, width: 0.53 mm; ex gall *Plagiotrochus razeti* on *Quercus ilex*, n = 11; Body length: 2.21 ± 0.46 mm (min-max: 1.80–3.40 mm), width: 1.10 ± 0.10 mm (min-max: 0.93–1.27 mm); ex gall *Trigonaspis mendesi* on *Quercus faginea*, n = 1; Body length: 1.55 mm, width: 0.80 mm.

The morphology of the terminal larva of this species is very similar to that of the *Ormyrus nitidulus* larva. The larvae of *Ormyrus pomaceus* from galls of *Andricus* and *Trigonaspis* species were distinguished from those of *Ormyrus nitidulus* by the following characters: integument of thoracic and abdominal segments with conspicuous blister-like sculpture; distance among vertex setae longer than the distance between antennae; *am* 0.47 as long as the distance between antennae; *lcs* situated above the level of *cs*, being separated from *cs* by 1.2-fold the distance between *cs*; and maxillary palps not visible (Table 2).

For the *Ormyrus pomaceus* larvae that inhabited *Plagiotrochus* galls (Figs 4C, 6B, 8C, and 10A), the differences between *Ormyrus pomaceus* ex *Andricus* and ex other host genera, such as *Trigonaspis* (Figs 4B, 6A, 8B, and 9H), were the following: *am* shorter in length than the separation between antennae (Table 2) and maxillary palps conspicuous.

##### Biology.

*Ormyrus pomaceus* is a polyphagous ectoparasitoid that attacks more than 56 different cynipid galls on *Quercus* trees (Figs 14F and I; 15D and E) (Askew et al. 2013). Nevertheless, results from ongoing unpublished molecular studies clearly indicate that *Ormyrus pomaceus* includes a complex of sibling or cryptic species that are segregated according to cynipid hosts, host plant species and ecological preferences (Hernandez Nieves et al. unpublished, Stone pers. comm.). On the Iberian Peninsula, among the most regular host species of *Ormyrus pomaceus*, we found the asexual generations of *Andricus grossulariae* (Fig. 14D and E) and *Andricus pictus* (Fig. 14G and H), *Trigonaspis mendesi* (Fig. 14J and K) and *Trigonaspis brunneicornis* (Figs 14L and 15A) on *Quercus pyrenaica* and *Quercus faginea* and the galls of asexual species of *Plagiotrochus* on *Quercus ilex* and *Quercus coccifera* (Fig. 15B and C).

#### Ormyrus rufimanus

Taxon classificationAnimaliaHymenopteraOrmyridae

Mayr, 1904.

##### Material examined.

ex gall *Xestophanes potentillae* on *Potentilla reptans*, Spain, Madrid: Cotos de Monterrey, 24.VI.03, J. L. Nieves leg (n = 2); Madrid: Villalvilla, 26.VIII.05, J. L. Nieves leg (n = 9); Madrid: Villar del Olmo, 03.X.04, J. L. Nieves leg (n = 23); Tarragona: Colldejou, 14.VIII.03, J. L. Nieves leg (n = 7).

##### Description.

n = 41; Body length: 1.69 ± 0.39 mm (min-max: 1.13–2.53 mm), width: 0.84 ± 0.22 mm (min-max: 0.47-1.27). Body fusiform, abdominal segments tapering abruptly towards ANS (Figs 4D, 6C); *adp* strongly remarked; integument of abdominal segments smooth but with blister-like sculpture extended in part of thoracic segments; setae on thoracic segment long, not longer than length of a thoracic segment, shorter on abdominal region. Head 1.03 as wide as high (Fig. 8D); integument on the face smooth; vertex concave; *an* situated at mid position in the face; vertex setae equally separated than the distance between antennae; *am* situated at the same level of *an*; *am* short, 0.22 times as long as the separation between antennae (Table 2). On clypeus *lcs* situated at the same level of *cs*, both equal in length (Fig. 10B); lateral lobes of labrum slightly differentiated and almost fused with the medial lobe; posterior margin of medial lobe of labrum straight; mandibles unidentated; tooth apex acute (Table 2).

##### Biology.

This species is extremely host-specific and is exclusively associated with galls on the runners and roots of *Potentilla reptans* (Rosaceae) induced by *Xestophanes potentillae* (Retzius) (Fig. 15H and I) (Askew et al. 2006). On the Iberian Peninsula, two forms of the galls were found. One form was on stems or runners close to or beneath the soil surface that consisted of round swellings (Fig. 15G), and the others formed on subterranean rhizomes (Fig. 15F).

In the first stages, the larva of *Ormyrus rufimanus* and the paralyzed host larva co-occurred; in later stages, the remains of the host larva appeared on the ventral surface of the *Ormyrus rufimanus* larva. In dissected galls, the larvae of *Ormyrus rufimanus* were extracted from irregularly shaped larval gall cells, which indicated that vegetal material was consumed at the terminal larval stage, as observed with other *Ormyrus* species such as *Ormyrus papaveris*. Based on additional observations, we found larvae of *Eupelmus vesicularis* (Chalcidoidea, Eupelmidae) were hyperparasitoids of *Ormyrus rufimanus* pupae.

#### Ormyrus wachtli

Taxon classificationAnimaliaHymenopteraOrmyridae

Mayr, 1904.

##### Material examined.

ex gall *Neaylax verbenacus* on *Salvia verbenaca*, Spain, Madrid: Dehesa de Arganda, 09.VI.02 J. L. Nieves leg (n = 1).

##### Description.

n = 1; Body length: 1.67 mm, width: 0.80 mm. The larva of this species is similar to the larva of *Ormyrus diffinis*, from which may be distinguissed as follows: body fusiform, wider at the level of segments ABS2-ABS3, tapering progresively towards ANS; anal segment wider tan length; *adp* absent; integument of the abdominal and thoracic segments blister-like. Head 1.18 as wide as high (Fig. 8E) with blister-like sculpture extended on all the head; antennae situated at mid position in the face; *ams* situated clearly above the antennae; lateral lobes of labrum almost fused with the medial lobe; ventral margin of medial lobe of labrum straight (Fig. 10C).

##### Biology.

The larva of *Ormyrus wachtli* is a solitary ectoparasitoid of larvae of cynipids, inducing galls on fruits of *Salvia* (Lamiaceae). Along the Iberian Peninsula and in southern Europe, the species is associated with galls of *Neaylax salviae* (Giraud) on *Salvia lavandulifolia* (Fig. 15L) and *Neaylax verbenacus* (Nieves-Aldrey) on *Salvia verbenaca* (Fig. 15J and K) (Nieves-Aldrey 2001, Nieves-Aldrey and Askew 2002, Askew et al. 2006). The species has a bivoltine life cycle.

## Discussion

As discussed in published studies on other families of Chalcidoidea (Gómez et al. 2008, 2011, 2013; Gómez and Nieves-Aldrey 2012, 2017; Nieves-Aldrey et al. 2008), the larval characters have potential value in systematic and phylogenetic studies of the group. Moreover, the taxonomy and identification of Chalcidoidea associated with gall-inducing insects is more robust when data on larval morphology of the species are available. In this work, for the first time, the primary morphological traits of larvae of *Ormyrus* species and their potential value in the systematics of the family Ormyridae of the Chalcidoidea is discussed.

### Terminal-instar larval morphology and *Ormyrus* taxonomy

The larvae of Ormyridae have a combination of traits that differentiate this family from other related chalcidoid families with a similar lifestyle as parasitoids of gall-inducer insects. Compared with larvae of Torymidae or Pteromalidae (Gómez et al. 2008; Gómez and Nieves-Aldrey 2012), the body setae are relatively short, the abdominal segments are particularly inconspicuous, and the labrum is normally divided into three lobes, with two laterals and one larger, central.

Nevertheless, larvae of Ormyridae resemble those of Eurytomidae in the relative length of body setae, with the thoracic setae relatively longer than those of the abdominal segments (Gómez et al. 2011, 2013). However, the larvae of Ormyridae have a single-toothed mandible, whereas the larval mandibles of eurytomids are bidentate. Additionally, the labrum of Ormyridae larvae is typically undivided or only has three lobes, whereas that of Eurytomidae larvae is usually divided into five lobes. For many characters, the larva of Ormyridae also resembles the larva of Torymidae and Eupelmidae (Gómez et al. 2008; Nieves-Aldrey et al. 2008; Gómez and Nieves-Aldrey 2017), although some conspicuous traits permit easy differentiation. First, the ormyrid larvae differ from torymids because of the much shorter abdominal body setae and the lower number of cephalic setae. Second, the larvae of Eupelmidae are easily distinguished from those of Ormyridae by the ventral margin of the clypeus, which is regularly serrate in eupelmid larvae and entire in ormyrid larvae. Finally, Ormyridae are easily distinguished from other chalcidoid parasitoids of galls, such as Pteromalidae and Eulophidae because the terminal-instar larvae of these two families are essentially glabrous (Gómez and Nieves-Aldrey 2012, 2017).

### Species differentiation and relationships related to terminal-instar larval characters

Based on unpublished results of combined morphological and molecular data, three primary clades defined the phylogenetic relationships of European species of *Ormyrus*, which were mostly congruent with host gall and plant data (Hernández Nieves 2007, Hernández Nieves et al. unpublished). The first clade was composed of the *Ormyrus* species that are parasitoids of oak gall wasps (tribe Cynipini), with one species, *Ormyrus rufimanus*, associated with cynipid galls on *Xestophanes* (Rosaceae) (tribe Diastrophini). The second clade was composed of *Ormyrus* species that attack cynipid gall wasps on herbs tribes Aulacideini, Aylacini and Phanacidini (Ronquist et al. 2015). The third clade contained the two *Ormyrus* species, *Ormyrus cupreus* and *Ormyrus orientalis*, that attack non-cynipid hosts.

The presence or absence of a blister-like sculpture was one larval feature that was moderately congruent with this division. Although the terminal-instar larvae of the *Ormyrus* species that are parasitoids of herb gall wasps did not present the blister-like sculpture, the sculpturing was found in the species associated with oak gall wasps (Cynipini) and in *Ormyrus cupreus*, the species associated with eurytomid galls. However, *Ormyrus wachtli* and *Ormyrus rufimanus* were exceptions, although the blister-like sculpture was found on the thorax of *Ormyrus rufimanus*. For *Ormyrus wachtli*, the conspicuous blister-like sculpture is one of the distinctive diagnostic characters, in combination with a pair of supraclypeal setae, which is absent in the other species.

Identification of the larvae of *Ormyrus* species is usually relatively easy based on their host and plant specificity. Nevertheless, for polyphagous species, such as the complex of *Ormyrus pomaceus* and *Ormyrus nitidulus* associated with cynipid galls on *Quercus* and those species that share hosts, the identification is more difficult. In many cases, the relative size of the larvae of the two species is a useful diagnostic tool because the larvae of *Ormyrus nitidulus* always exceeded 3 millimetres in size and the blister-like sculpture was not as conspicuous as with *Ormyrus pomaceus*. The preference of *Ormyrus nitidulus* for occupying the central cell of the gall, as a primary parasitoid of their hosts, was another useful trait to separate these two species. By contrast, the larvae of *Ormyrus pomaceus* may parasitize inducer and lethal inquilines, both from the genus *Synergus*, which occupy secondary larval chambers inside the galls.

The morphological characters described in this work on the systematics of terminal-instar larvae of Ormyridae were constant across species within the genus *Ormyrus*. The differences among species were not marked, although some small differences in morphological traits allowed the separation of some species or groups of species. We consider the data presented in this study to be a preliminary contribution to increasing information on the immature stages of one of the less studied families of Chalcidoidea. Therefore, the study must be expanded to include larvae of species from other zoogeographical regions and larvae of those species with biological traits different from that of the European species, such as the species associated with fig wasps on *Ficus* in tropical areas.

### Biological traits

Ormyridae are cosmopolitan inhabitants of different ecosystems worldwide, although the highest diversity is reported in Holarctic and Australasian regions, with only a few species cited from other zoogeographic regions such as Afrotropical and Neotropical, but these few species are likely a function of a lack of revisions of these faunas (Noyes 2016).

With reference to the diversity of the genus *Ormyrus* in the Palaearctic region, the 34 species recorded cover a wide range of insect host species, all of which are associated with different types of galls (Hernández Nieves 2007, Lotfalizadeh et al. 2012), primarily Cynipidae (Hymenoptera) (Hanson 1992) but also Tephritidae and Cecidomyiidae and Agromyzidae/Lonchaeidae (Diptera) (Bouček 1986). The exact roles of *Ormyrus* within specific gall communities remain unknown, but all species are idiobiont ectoparasitoids or hyperparasitoids, even the few Afrotropical and Australasian species, some of which are obligate or facultative parasitoids on fig wasp communities in *Ficus* plants (van Noort et al. 2007). Moreover, many of the undescribed African *Ormyrus* species are associated with shrub galls on a variety of plant taxa (S. van Noort, pers. comm.).

Of the ten European species examined in this study, eight species formed part of the parasitoid community associated with cynipid galls, and two were associated with gall tephritids and gall eurytomids. With reference to the species associated with gall wasps, as with the related Torymidae (Gómez et al. 2008), remarkably, most are parasitoids with a narrow host range. Whereas monophagy is apparently common within the parasitoid community associated with herb gall wasps (tribes Aylacini and Aulacideini), polyphagous species are more common in parasitoid communities associated with galls on *Quercus* species (cynipid species included in the tribe Cynipini) (Askew et al. 2006, 2013).

Some of the ormyrid species that we studied were dominant in the parasitoid communities associated with their host galls. For example, *Ormyrus papaveris* was the most abundant parasitoid species in galls on poppy heads induced by *Aylax papaveris*. Similarly, *Ormyrus gratiosus* (attacking *Isocolus scabiosae*) and *Ormyrus diffinis* and *Ormyrus wachtli* (attacking galls of *Liposthenes kerneri* on *Nepeta* and *Neaylax* ssp. on *Salvia*, respectively) are the most abundant parasitoid species in those gall parasitoid communities (Askew et al. 2006, Hernández et al. unpublished). *Ormyrus wachtli* has a co-dominant relationship with *Eurytoma infracta* (Eurytomidae), which are the only known parasitoids in galls of *Neaylax verbenacus* to date and are responsible for more than 80% of gall parasitism in all geographical locations.

*Ormyrus cupreus* occupies a unique position within the parasitoid community of *Eurytoma gallephedrae* (Askew and Blasco-Zumeta 1998). In galls of this eurytomid species on *Ephedra nebrodensis*, the dominant species is *Brasema ephedricola* (Eupelmidae). *Ormyrus cupreus* is recorded as a hyperparasitoid of *Eupelmus* sp. within the community (Askew and Blasco-Zumeta 1998), and cannibalistic behaviour has been observed among larvae of *Ormyrus cupreus*.

According to previous molecular and morphological phylogenetic analyses (Hernández Nieves 2007), the evidence is strong that *Ormyrus pomaceus* includes a complex of cryptic or sibling species. Within this complex, we identified at least three different groups, based on morphological, molecular and biological data. The “*plagiotrochus*” group consisted of *Ormyrus pomaceus* associated with *Plagiotrochus* cynipid-galls on *Quercus* trees section *Ilex* (specifically *Quercus ilex* and *Quercus coccifera*) and *Ormyrus pomaceus* specimens reared from asexual galls of *Plagiotrochus razeti* (Fig. 15B), with these galls also found on runners of *Quercus ilex*. The terminal-instar larva of Ormyrus pomaceus f. plagiotrochus is a solitary parasitoid that usually occupied the cynipid gall chamber, which was seldom deformed secondarily (Fig. 15D and E). We also found larvae of *Ormyrus pomaceus* in galls of *Plagiotrochus fusifex* on *Quercus coccifera* (Fig. 15C).

The “*trigonaspis*” group was composed of *Ormyrus pomaceus* “*sensu lato*” specimens that attacked small leaf galls induced by species of *Trigonaspis*, a genus which is circumscribed primarily on the Iberian Peninsula and is represented by at least three endemic species: *Trigonaspis mendesi*, *Trigonaspis brunneicornis* and *Trigonaspis baeticus* (Nieves-Aldrey 2001). Individuals of *Ormyrus pomaceus* in this group were relatively abundant in galls of *Trigonaspis mendesi* (Fig. 14J and K) on *Quercus faginea* and *Trigonaspis brunneicornis* on *Quercus pyrenaica* (Figs 14L and 15A).

The “*pomaceus*” *sensu stricto* group was composed of the core individuals of *Ormyrus pomaceus* reared from the other cynipid-galls on several species of *Quercus*, with approximately 84 different cynipid host gall species recorded (Askew et al. 2013), excluding the species of *Plagiotrochus* and *Trigonaspis* (Fig. 14D, E, F, G, H, and I).

The larvae of *Ormyrus pomaceus* *sensu stricto* were located in dissected galls of asexual generations of *Andricus pictus* (Fig. 14G, H, and I) and *Andricus grossulariae* (Fig. 14D, E, and F), and the larvae are either parasitoids of the cynipid larvae or the gall maker or the lethal inquiline of the genus *Synergus*.

The community of ormyrid parasitoids of cynipid galls is usually composed of solitary ectoparasitoids. Notably, in this work, we reported some cases of secondary phytophagy in several ormyrid species. For example, the larvae of *Ormyrus papaveris* were observed moving inside the gall cell and touching the walls with their mandibles, which was similar to the behaviour of *Ormyrus rufimanus*; as a result, the gall cells became larger and deformed. Larval phytophagy has been described for some species of Eurytomidae (Crosby 1909; Bugbee 1941; Zerova 1981, 1993; Bouček 1988; Henneicke et al. 1992; Dawah and Rothfritz 1996; Askew and Blasco-Zumeta 1998; La Salle 2005; Gómez et al. 2011, 2013), but this behaviour had not been previously recorded in the Ormyridae.

## Conclusions

The external morphology of final instar ormyrid larvae has been documented and the potential use of the characters in the taxonomy and systematics of this poorly studied Chalcidoidea group explored. Our data will assist in the reliable identification of the species of this chalcidoid family during studies of cynipid gall communities and food webs in which the accurate identifications of species are of great importance. However, much further work is required, including investigations of a wider selection of ormyrid species and descriptions of the other immature stages, in addition to more detailed observations of their parasitic behaviour.

## Supplementary Material

XML Treatment for Ormyrus capsalis

XML Treatment for Ormyrus cupreus

XML Treatment for Ormyrus diffinis

XML Treatment for Ormyrus gratiosus

XML Treatment for Ormyrus nitidulus

XML Treatment for Ormyrus orientalis

XML Treatment for Ormyrus papaveris

XML Treatment for Ormyrus pomaceus

XML Treatment for Ormyrus rufimanus

XML Treatment for Ormyrus wachtli

## Supplementary materials

Supplementary material 1 Characters of *Ormyrus* larvae used for systematic study Data type: species data Explanation note: Characters are listed by body region, for the body, head and under lip complex, which for the latter is subdivided between labrum, maxillae and mandibles. This dataset is made available under the Open Database License (http://opendatacommons.org/licenses/odbl/1.0/). The Open Database License (ODbL) is a license agreement intended to allow users to freely share, modify, and use this Dataset while maintaining this same freedom for others, provided that the original source and author(s) are credited.Jose F. Gómez, María Hernández Nieves, Severiano F. Gayubo, Jose Luis Nieves-Aldrey

Supplementary material 2 Character states of *Ormyrus* larvae included in the systematic study Data type: species data Explanation note: Observed character states of characters listed in Appendix 1. Explanation of symbols: monomorphic states 0–3; not applicable (*); unknown (?). Characters are unordered. This dataset is made available under the Open Database License (http://opendatacommons.org/licenses/odbl/1.0/). The Open Database License (ODbL) is a license agreement intended to allow users to freely share, modify, and use this Dataset while maintaining this same freedom for others, provided that the original source and author(s) are credited.Jose F. Gómez, María Hernández Nieves, Severiano F. Gayubo, Jose Luis Nieves-Aldrey

## References

[B1] Aguiar AP Deans AR Engel MS Forshage M Huber JT Jennings JT Johnson NF Lelej AS Longino JT Lohrmann V Mikó I Ohl M Rasmussen C Taeger A Yu DSK (2013) Order Hymenoptera. In: Zhang ZQ (Ed.) Animal biodiversity: an outline of higher-level classification and survey of taxonomic richness (Addenda 2013). Zootaxa 3703(1), 51–62. 10.11646/zootaxa.3703.1.1 26146682

[B2] Askew RR (1966) Observations on the British species of *Megastigmus* Dalman (Hym. Torymidae) which inhabit cynipid oak galls. Entomologist 99: 124–128.

[B3] Askew RR (1994) Two new European species of *Ormyrus* (Hym. Ormyridae). Entomologist’s Monthly Magazine 130: 89–90.

[B4] Askew RR Blasco-Zumeta J (1998) Insects associated with galls of a new species of Eurytomidae (Hymenoptera: Chalcidoidea) on *Ephedra nebrodensis* in Spain. Journal of Natural History 32: 805–809. https://doi.org/10.1080/00222939800770431

[B5] Askew RR Plantard O Gómez JF Hernández-Nieves M Nieves-Aldrey JL. (2006) Catalogue of parasitoids and inquilines in galls of Aylacini, Diplolepidini and Pediaspidini (Hym., Cynipidae) in the West Palaeartic. Zootaxa 1301: 1–60.

[B6] Askew RR Melika G Pujade-Villar J Schonrogge K Stone GN Nieves-Aldrey JL (2013) Catalogue of parasitoids and inquilines in cynipid oak galls in the West Palaearctic. Zootaxa 3643: 1–133. https://doi.org/10.11646/zootaxa.3643.1.1 25340198 10.11646/zootaxa.3643.1.1

[B7] Bouček Z (1970) Contribution to the knowledge of Italian Chalcidoidea based mainly on a study at the Institute of Entomology in Turin, with descriptions of some new European species (Hymenoptera). Memorie della Società Entomologica Italiana 49: 35–102.

[B8] Bouček Z (1977) A faunistic review of the Yugoslavian Chalcidoidea (Parasitic Hymenoptera). Acta Entomologica Jugoslavica 13 (Supplement): 1–145.

[B9] Bouček Z (1986) Taxonomic study of chalcidoid wasps (Hymenoptera) associated with gall midges (Diptera: Cecidomyiidae) on mango trees. Bulletin of Entomological Research 76(3): 393–407. https://doi.org/10.1017/S0007485300014899

[B10] Bouček Z (1988) Australasian Chalcidoidea (Hymenoptera). A Biosystematic Revision of Genera of fourteen Families, with a Reclassification of Species. CAB International, Wallingford, 838 pp.

[B11] Bouček Z Watsham A Wiebes JT (1981) The fig wasp fauna of the receptacles of *Ficus thonningii* (Hymenoptera, Chalcidoidea). Tijdschrift voor Entomologie 124: 149–233.

[B12] Bugbee R (1941) A new species of the *Eurytoma rhois* complex from the seeds of Schmaltzia (Rhus) trilobata. Journal of the Kansas Entomological Society 14: 97–102.

[B13] Clausen CP (1940) Entomophagous Insects. McGraw Hill Book Co., Inc., New York, 688 pp.

[B14] Crosby C (1909) On certain seed-infesting chalcid flies. Cornell University. Agricultural Experiment Station Bulletin 256: 367–388.

[B15] Cutler JR (1955) The morphology of the head of the final instar larva of *Nasonia vitripennis* Walker (Hymenoptera: Chalcidoidea). Proceedings of The Royal Entomological Society of London (A) 30(4-6): 73–81. https://doi.org/10.1111/j.1365-3032.1955.tb00181.x

[B16] Dawah HA Rothfritz H (1996) Generic-level identification of final instar larvae of *Eurytoma* and their parasitoids associated with grasses (Poaceae) in N.W. Europe (Hymenoptera: Braconidae, Eulophidae, Eupelmidae, Eurytomidae, Ichneumonidae, Pteromalidae). Journal of Natural History 30: 1517–1526. https://doi.org/10.1080/00222939600770861

[B17] Doğanlar M (1984) Notes on Chalcidoidea of Turkey. I. Chalcididae, Eurytomidae, Torymidae, Ormyridae, Perilampidae, Eucharitidae. Türkiye Bitki Koruma Dergisi 8(3): 151–158.

[B18] Doğanlar M (1991a) Systematic positions of some taxa in Ormyridae and descriptions of a new species in *Ormyrus* from Turkey and a new genus in the family. Turkiye entomoloji Dergisi 15: 1–13.

[B19] Doğanlar M (1991b) Systematic studies on the species of *Cyrtosoma* Perris from Turkey and descriptions of some new species (Hym.: Ormyridae). Turkiye entomoloji Dergisi 15: 71–87.

[B20] Erdös J (1946) Genere nova et species novae chalcidoidarum (Hym.). Annales Historico-Naturales Musei Nationalis Hungarici 39: 131–165.

[B21] Gómez JF Nieves-Aldrey JL (2012) Notes on the larval morphology of Pteromalidae (Hymenoptera: Chalcidoidea) species parasitoids of gall wasps (Hymenoptera: Cynipidae) in Europe. Zootaxa 3189: 39–55.

[B22] Gómez JF Nieves-Aldrey JL (2017) Terminal-instar larval morphology and systematics of Eulophidae and Eupelmidae species (Hym., Chalcidoidea) parasitoids of gall wasps (Hym., Cynipidae) communities from Europe. Insect Systematics and Evolution 48: 1–28.

[B23] Gómez JF Nieves-Aldrey JL Hernández Nieves M (2008) Comparative morphology, biology and phylogeny of terminal-instar larvae of the European species of Toryminae (Hym., Chalcidoidea, Torymidae) parasitoids of gall wasps (Hym. Cynipidae). Zoological Journal of the Linnean Society 154: 676–721. https://doi.org/10.1111/j.1096-3642.2008.00423.x

[B24] Gómez JF Nieves-Aldrey JL Hernández Nieves M Stone GN (2011) Comparative morphology and biology of terminal instar larvae of some *Eurytoma* (Hymenoptera, Eurytomidae) species parasitoids of gall wasps (Hymenoptera, Cynipidae) in Western Europe. Zoosystema 33(3): 287–323. https://doi.org/10.5252/z2011n3a3

[B25] Gómez JF Nieves-Aldrey JL Stone GN (2013) On the morphology of the terminal-instar larvae of some European species of *Sycophila* (Hymenoptera, Eurytomidae) parasitoids of gall wasps (Hymenoptera, Cynipidae). Journal of Natural History 47(47–48): 2937–2960. https://doi.org/10.1080/00222933.2013.791937

[B26] Hanson P (1992) The Nearctic species of *Ormyrus* Westwood (Hymenoptera: Chalcidoidea: Ormyridae). Journal of Natural History 26(6): 1333–1365. https://doi.org/10.1080/00222939200770761

[B27] Henneicke K Dawah HA Jervis MA (1992) Taxonomy and biology of the final-instar larvae of some Eurytomidae (Hymenoptera: Chalcidoidea) associated with grasses in the UK. Journal of Natural History 26: 1047–1087. https://doi.org/10.1080/00222939200770621

[B28] Hernández Nieves M (2007) Taxonomía, biología y filogenia de las especies ibéricas de Ormyridae. Tesis Doctoral. Universidad de Salamanca, Salamanca, 348 pp.

[B29] La Salle J (2005) Biology of gall inducers and evolution of gall induction in Chalcidoidea (Hymenoptera: Eulophidae, Eurytomidae, Pteromalidae, Tanaostigmatidae, Torymidae). In: Raman A Schaeffer CW Withers TM (Eds) Biology, Ecology and evolution of Gall-inducing Arthropods. Science Publishers, Inc., Enfield, New Hampshire, 503–533.

[B30] Lotfalizadeh H Askew RR Fuentes-Utrilla P Tavakoli M (2012) The species of *Ormyrus* Westwood (Hymenoptera: Ormyridae) in Iran with description of an unusual new species. Zootaxa 3300: 34–44.

[B31] Narendran TC (1999) Indo-Australian Ormyridae (Hymenoptera: Chalcidoidea). Privately published, University of Calicut, India, 227 pp.

[B32] New TR (2012) Hymenoptera and conservation. Wiley-Blackwell, Chichester, 218 pp https://doi.org/10.1002/9781118381250

[B33] Nieves-Aldrey JL (1984) Primeros datos de los representantes de la familia Ormyridae en España, con la descripción de una nueva especie (Hymenoptera, Chalcidoidea). Graellsia 40: 119–128.

[B34] Nieves-Aldrey JL (2001) Hymenoptera, Cynipidae. In: Ramos MA et al. (Eds) Fauna Ibérica, vol. XVI Museo Nacional de Ciencias Naturales (CSIC), Madrid, 636 pp.

[B35] Nieves-Aldrey JL Askew RR (2002) Calcídidos (Hym., Chalcidoidea) asociados a agallas de Aylacini y Diplolepidini (Hym., Cynipidae) en España. Boletín de la Asociación española de Entomología 26(1–2): 11–37.

[B36] Nieves-Aldrey JL Vårdal H Ronquist F (2005) Comparative morphology of terminal-instar larvae of Cynipoidea: phylogenetic implications. Zoologica Scripta 34: 15–36. https://doi.org/10.1111/j.1463-6409.2005.00175.x

[B37] Nieves-Aldrey JL Hernández Nieves M Gómez JF (2007) A new afrotropical species of *Ormyrus* Westwood, 1832 (Hymenoptera, Chalcidoidea, Ormyridae). Graellsia 63(1): 53–60. https://doi.org/10.3989/graellsia.2007.v63.i1.80

[B38] Nieves-Aldrey JL Hernández Nieves M Gómez JF (2008) Larval morphology and biology of three European *Megastigmus* (Hymenoptera, Torymidae, Megastigminae) parasitoids of gall wasps, including a comparison with three larvae of two seed-infesting species. Zootaxa 1746: 46–60.

[B39] Noyes JS (2016) Universal Chalcidoidea Database. World Wide Web electronic publication http://www.nhm.ac.uk/chalcidoids

[B40] Parker HL (1924) Recherches sur les formes post-embryonnaires des chalcidiens. Annales de la Société Entomologique de France 93: 262–379.

[B41] Parker HL Thompson WR (1925) Notes on the larvae of the Chalcidoidea. Annals of the Entomological Society of America 18: 384–395. https://doi.org/10.1093/aesa/18.3.384

[B42] Rasplus J-Y La Salle J Delvare G McKey D Webber B (2011) A new afrotropical genus and species of Tetrastichinae (Hymenoptera: Eulophidae) inducing galls on *Bikinia* (Fabaceae: Caesalpinioideae) and a new species of *Ormyrus* (Hymenoptera: Ormyridae) associated with the gall. Zootaxa 2907: 51–59.

[B43] Rivosecchi L (1958) *Eurytoma tristis* Mayr e *Ormyrus hungaricus* Erdös (Hymenoptera, Chalcididae). Bolletino de Zoología Agraria e Bachicultura Milano 1: 187–208.

[B44] Sellenschlo U Wall I (1984) The chalcidoid wasps of central Europe. In: Verlag Erich Bauers, Keltern (Eds) Systematic, biology and bibliography of the Torymidae and Ormyridae. German Federal Republic, 111 pp.

[B45] Sharkey MJ Fernandez F (2006) Biología y diversidad de hymenoptera. In: Fernández F Sharkey MJ (Eds) Introducción a los Hymenoptera de la Región Neotropical. Sociedad Colombiana de Entomología Universidad Nacional de Colombia, Bogotá, 93–113.

[B46] Short JRT (1952) The morphology of the head of larval Hymenoptera with special reference to the head of the Ichneumonoidea, including a classification of the final instar larvae of the Braconidae. Transactions of the Royal Entomological Society of London 103(2): 27–84. https://doi.org/10.1111/j.1365-2311.1952.tb02262.x

[B47] Shorthouse JD (1972) An emergence technique for small insects. The Canadian Entomologist 104: 1331–1332. https://doi.org/10.4039/Ent1041331-9

[B48] Tutin TG Heywood VH Burges NA Valentine DH Moore DM (1980) Flora Europaea (5 vol). Cambridge University Press, Cambridge, 506 pp.

[B49] van Noort S (2004) Fig wasp (Hymenoptera: Chalcidoidea: Agaonidae, Pteromalidae, Eurytomidae and Ormyridae) and *Ficus* (Moraceae) species richness and biogeography of Monts Doudou in southwestern Gabon. California Academy of Sciences Memoir 28: 217–233.

[B50] van Noort S Stone GN Whitehead VB Nieves-Aldrey JL (2007) Biology and redescription of *Rhoophilus loewi* (Cynipidae: Cynipoidea: Hymenoptera), with evolutionary implications on the inquilinism in gall wasps. Biological Journal of the Linnean Society 90: 153–172. https://doi.org/10.1111/j.1095-8312.2007.00719.x

[B51] Vance AM Smith HD (1933) The larval head of Parasitic Hymenoptera and nomenclature of its parts. Annals of Entomological Society of America 26: 86–94. https://doi.org/10.1093/aesa/26.1.86

[B52] Vårdal H Gómez JF Nieves-Aldrey JL (2016) Ovarian egg morphology in chalcidoid wasps (Hymenoptera: Chalcidoidea) parasitizing gall wasps (Hymenoptera: Cynipidae). Graellsia 72(1): e044 https://doi.org/10.3989/graellsia.2016.v72.165

[B53] Yao YX Yang ZQ (2004) A new species of Ormyridae (Hymenoptera: Chalcidoidea) parasitizing a gall-making weevil on twigs of the bunge hackberry tree in China. Entomologica Fennica 15(3): 142–148.

[B54] Zerova MD (1981) Phytophagous species of *Eurytoma* Illiger (Hymenoptera, Chalcidoidea, Eurytomidae) that develop in seeds of certain Cruciferae. Vestnik Zoologii 2: 80–82.

[B55] Zerova MD (1993) The new group of phytophagous chalcids of the family Eurytomidae (Hymenoptera, Chalcidoidea). Zoologicheskiy Zhurnal 72(10): 68–74.

[B56] Zerova MD (1995) A new species of the genus *Eurytoma* (Hymenoptera, Eurytomidae) from Thailand. Zhurnal Ukrayns’kogo Entomologichnogo Tovaristva 1(3–4): 59–62.

[B57] Zerova MD Seryogina LY (2006) Review of Paleartic Ormyridae (Hymenoptera, Chalcidoidea), with Description of Two New Species. Vestnik Zoologii 40(1): 27–40.

[B58] Zerova MD Seryogina LY (2014a) A new species of the genus *Ormyrus* Westwood (Hymenoptera: Ormyridae) from the Russian Far East. Proceedings of the Russian Entomological Society St. Petersburg 85(1): 188–190.

[B59] Zerova MD Seryogina LY (2014b) A new species of the genus *Ormyrus* (Hymenoptera, Ormyridae) from the steppe zone of Ukraine. Vestnik Zoologii 48(3): 281–283. https://doi.org/10.2478/vzoo-2014-0032

[B60] Zerova MD Seryogina LY van Harten A (2012) New and formerly unknown Ormyridae species from the United Arab Emirates (Hymenoptera, Chalcidoidea). Vestnik Zoologii 46(2): 113–121. https://doi.org/10.2478/v10058-012-0011-3

[B61] Zerova MD Seryogina LY Kuslitzkyb WS Argov Y (2015) New species of the Chalcidoidea wasps of the genus *Ormyrus* (Hymenoptera, Ormyridae) from Israel. Entomological Review 95(4): 507–510. https://doi.org/10.1134/S0013873815040132

