# Supplementary material for: Terminal-instar larval systematics and biology of west European species of Ormyridae associated with insect galls (Hymenoptera, Chalcidoidea)
Source: Zookeys. 2017 Jan 10;(644):51–88. doi: 10.3897/zookeys.644.10035 (PMC5242259; doi:10.3897/zookeys.644.10035)
Supplement: Supplementary material 1 — Characters of Ormyrus larvae used for systematic study [file zookeys-644-051-s001.doc]

APPENDIX 1

###### Characters of Ormyrus larvae used for systematic study.

###### Characters are listed by body region, for the body, head and under lip complex, which for the latter is subdivided between labrum, maxillae and mandibles.

###### LARVAL BODY

1. Body shape in ventral view; relation between width and length at the level of 3rd abdominal segment: (0) >3.0, relatively long and narrow (outgroup: Torymidae); (1) between 2.5-3.0 (outgoups: Eupelmidae, Pteromalidae); (2) between 2.1-2.5 abdominal segments abruptly tapering towards the anal segment (Fig. 4A); (3) <2.1, body relatively short and wide, but not abruptly tapering towards the anal segment (Fig. 3A).

2. Anterodorsal protuberances (lateral view): (0) absent, the intersegmental membranes not protruding (Fig 5D); (1) present, the intersegmental membranes of body segments 1-6 slightly protruding (Fig. 5A); (2) present, the intersegmental membranes of body segments 1-6 strongly protruding (Fig. 5A).

3. Body setae (lateral view): (0) absent; body glabrous without any conspicuous setae (outgroup: Pteromalidae); (1) generally only three rows of setae at the level of thoracic segments (dorsal, lateral and ventral row); setae shorter than the length of one segment (Figs. 5, 6); (2) several rows of long setae in all body segments, generally denser in ventral region and almost as long as the length of one segment (outgroup: Torymidae).

4. Relative length of thoracic segments (lateral view): (0) long, at least as long as the length of one segment (outgroups: Eupelmidae, Eurytomidae, Torymidae); (1) short, relatively shorter than the length of one segment (Figs. 5, 6).

5. Relative length of abdominal setae (lateral view): (0) long, at least as long as the length of one segment (outgroup: Torymidae); (1) relatively long, at least as long as the half length of one segment measured at the spiracles level (outgroup: Eupelmidae); (2) very short, much shorter than the half length of one segment measured at the spiracles level (Figs. 5, 6).

6. Abdominal segments (lateral view): (0) smooth, without sculpture (Fig. 5A); (1) pustulate with blister-like swellings (Fig. 5B).

7. Thoracic segments (lateral view): (0) smooth, without sculpture (Fig. 5A); (1) pustulate with blister-like swellings at least partially (Figs. 5B, 7B, 8D); (2) irregular blister-like sculpture (outgroup: Eupelmidae).

HEAD AND MOUTH PARTS

8. Shape of head: (0) more or less rounded, slightly broader than high (outgroup: Pteromalidae); (1) more or less trapezoid, clearly broader than high (Figs. 7, 8).

9. Head integument (anterior view): (0) smooth, without sculpture (Fig. 7A); (1) pustulate with blister-like swellings only in genal area (Fig. 7D); (2) pustulate with blister-like swellings fully extended for the head (Fig. 7B).

10. Upper margin of vertex: (0) slightly concave or medially incised (Fig. 7C); (1) straight (Fig. 8A); (2) convex (Fig. 7F).

11. Relative position of antennae on head (anterior view): (0) situated more or less midway between upper margin of vertex and the clypeus; relation between both distances, (upper vertex and anterior margin of clypeus to antennae) and the antennae = 0.6-1.3 (Figs. 7, 8); (1) situated relatively high on the upper face; much closer to the anterior margin of vertex than to the clypeus; relation > 1.3 (outgroup: Pteromalidae).

12. Setae of head (anterior view): (0) setae very short, inconspicuous or absent (outgroup: Pteromalidae); (1) basic pattern composed by not more than 5-7 pairs of setae always present (Figs. 7, 8).

13. Setae on vertex: (0) absent (outgroup: Pteromalidae); (1) one pair present (Figs. 7, 8); (2) two pair present (antero-lateral and antero-medial setae) (outgroup: Torymidae).

14. Relative position of antero-medial setae of antennal area: (0) situated at the same level or slightly above antennae (Fig. 7B); (1) clearly above the antennae in the upper face (Fig. 7D).

15. Ratio length of antero-medial setae of antennal area/ distance between antennae: (0) very short, < 0.1 the distance between antennae (outgroups: Pteromalidae, Torymidae); (1) short, < 0.3 the distance between antennae (Figs. 7E, 8A); (2) long, 0.3-0.7 the distance between antennae (Fig. 7D).

16. Distance between antero-lateral setae of vertex: (0) slightly shorter than the distance between antennae (outgroups: Eupelmidae, Eurytomidae, Torymidae); (1) as long as the distance between antennae (Fig. 7B); (2) clearly longer than the distance between antennae (Fig. 7A).

17. Supraclypeal setae: (0) absent (Fig. 7); (1) present, situated between clypeus and antennae (Fig. 8E).

18. Relative position of lateral-clypeal setae: (0) at the same level of clypeal setae (Fig. 9A); (1) slightly above the clypeal setae (Fig. 9B); (2) clearly above the clypeal setae (outgroup: Eurytomidae).

19. Length of the lateral-clypeal setae: (0) as long as clypeal setae (Figs. 9, 10); (1) longer than clypeal setae (outgroups: Eupelmidae, Torymidae).

20. Ventral margin of clypeus: (0) distinct (with a distinct suture) (Fig. 9C); (1) indistinct, the suture is not discernible (Fig. 9A).

21. Shape of clypeus: (0) straight and whole (Fig. 9C, E); (1) arcuate (outgroups: Pteromalidae, Torymidae); (2) straight and jagged (outgroup: Eupelmidae).

22. Two lateral lobes adjacent to the central piece of *labrum*: (0) absent (outgroups: Eupelmidae, Eurytomidae, Torymidae); (1) present but inconspicuous (Figs. 9A); (2) present and conspicuous (Figs. 9B)

23. Shape of the labrum: (0) subrectangular and undivided (outgroups: Eupelmidae, Pteromalidae, Torymidae); (1) divided into two lateral lobes and one rectangular central piece sometimes superficially subdivided into three lobes (Figs. 9, 10); (2) divided into two lateral lobes and one rectangular central piece strongly subdivided into five lobes (outgroup: Eurytomidae).

UNDER LIP COMPLEX

*Maxillae*

24. Maxillae: (0) indistinct, not well-differentiated from labium (outgroups: Pteromalidae, Torymidae); (1) well-differentiated from labium, triangular shaped (Figs. 9, 10).

25. Maxillary palps: (0) inconspicuous (outgroups: Eupelmidae, Pteromalidae, Torymidae); (1) conspicuous (Figs. 9, 10).

*Labium*

26. Labium: (0) convex, generally not collapsed (outgroups: Pteromalidae, Torymidae); (1) concave, generally not collapsed (Figs. 9, 10).

## *Mandibles*

27. Mandibles: (0) invisible externally, covered by labrum (Fig. 9A); (1) exposed in part, at least the tip being visible (Figs 9C, G; 10B).

28. Number of teeth: (0) one (Fig. 11); (1) two or more (outgroup: Eurytomidae).
