# Supplementary material for: Terminal-instar larval systematics and biology of west European species of Ormyridae associated with insect galls (Hymenoptera, Chalcidoidea)
Source: Zookeys. 2017 Jan 10;(644):51–88. doi: 10.3897/zookeys.644.10035 (PMC5242259; doi:10.3897/zookeys.644.10035)
Supplement: Supplementary material 2 — Character states of Ormyrus larvae included in the systematic study [file zookeys-644-051-s002.doc]

APPENDIX 2

Character states of *Ormyrus* larvae included in the systematic study

**Species** **Characters**

|  | **1** | **2** | **3** | **4** | **5** | **6** | **7** | **8** | **9** | **10** | **11** | **12** | **13** | **14** | **15** | **16** | **17** | **18** | **19** | **20** | **21** | **22** | **23** | **24** | **25** | **26** | **27** | **28** |
| --- | --- | --- | --- | --- | --- | --- | --- | --- | --- | --- | --- | --- | --- | --- | --- | --- | --- | --- | --- | --- | --- | --- | --- | --- | --- | --- | --- | --- |
| *Ormyrus capsalis* | 3 | 1 | 1 | 1 | 2 | 0 | 0 | 1 | 0 | 0 | 0 | 1 | 1 | 1 | 2 | 2 | 0 | 0 | 0 | 1 | * | 1 | 1 | 1 | 1 | 1 | 0 | 0 |
| *Ormyrus cupreus* | 2 | 2 | 1 | 1 | 2 | 1 | 1 | 1 | 2 | 0 | 0 | 1 | 1 | 0 | 1 | 1 | 0 | 1 | 0 | 1 | * | 2 | 1 | 1 | 1 | 1 | 0 | 0 |
| *Ormyrus diffinis* | 3 | 1 | 1 | 1 | 2 | 0 | 0 | 1 | 1 | 0 | 0 | 1 | 1 | 0 | 2 | 2 | 0 | 0 | 0 | 0 | 0 | 2 | 1 | 1 | 1 | 1 | 1 | 0 |
| *Ormyrus gratiosus* | 3 | 0 | 1 | 1 | 2 | 0 | 0 | 1 | 1 | 0 | 0 | 1 | 1 | 1 | 2 | 2 | 0 | 1 | 0 | 1 | * | 1 | 1 | 1 | 1 | 1 | 0 | 0 |
| *Ormyrus nitidulus* | 3 | 1 | 1 | 1 | 2 | 1 | 1 | 1 | 2 | 0 | 0 | 1 | 1 | 0 | 1 | 1 | 0 | 1 | 0 | 0 | 0 | 2 | 1 | 1 | 1 | 1 | 0 | 0 |
| *Ormyrus orientalis* | 3 | ? | 1 | 1 | 2 | 1 | 1 | 1 | 0 | 2 | 0 | 1 | 1 | 0 | 2 | 2 | 0 | 0 | 0 | 1 | * | 2 | 1 | 1 | 1 | 1 | 0 | 0 |
| *Ormyrus papaveris* | 2 | 2 | 1 | 1 | 2 | 0 | 0 | 1 | 0 | 1 | 0 | 1 | 1 | 1 | 1 | 2 | 0 | 0 | 0 | 1 | * | 1 | 1 | 1 | 1 | 1 | 1 | 0 |
| *Ormyrus pomaceus razeti* | 3 | 2 | 1 | 1 | 2 | 1 | 1 | 1 | 2 | 0 | 0 | 1 | 1 | 0 | 1 | 1 | 0 | 0 | 0 | 1 | * | 2 | 1 | 1 | 1 | 1 | 0 | 0 |
| *Ormyrus pomaceus mendesi* | 3 | 2 | 1 | 1 | 2 | 1 | 1 | 1 | 2 | 0 | 0 | 1 | 1 | 0 | 2 | 1 | 0 | 0 | 0 | 1 | * | 2 | 1 | 1 | 1 | 1 | 0 | 0 |
| *Ormyrus rufimanus* | 2 | 2 | 1 | 1 | 2 | 0 | 1 | 1 | 0 | 0 | 0 | 1 | 1 | 0 | 1 | 1 | 0 | 0 | 0 | 1 | * | 2 | 1 | 1 | 1 | 1 | 1 | 0 |
| *Ormyrus wachtli* | 2 | ? | 1 | 1 | 2 | 1 | 1 | 1 | 2 | 1 | 0 | 1 | 1 | 1 | 1 | 2 | 1 | 0 | 0 | 1 | * | 2 | 1 | 1 | 1 | 1 | 0 | 0 |
| *Eurytoma aspila* | 3 | 1 | 1 | 0 | 2 | 0 | 0 | 1 | 0 | 0 | 0 | 1 | 1 | 0 | 2 | 0 | 0 | 2 | 0 | 1 | * | 1 | 2 | 1 | 1 | 1 | 1 | 1 |
| *Torymus nobilis* | 0 | 0 | 2 | 0 | 0 | 0 | 0 | 1 | 0 | 2 | 0 | 1 | 2 | 1 | 0 | 0 | 1 | 1 | 1 | 0 | 1 | 0 | 0 | 0 | 0 | 0 | 0 | 0 |
| *Eupelmus cerris* | 1 | 2 | 1 | 0 | 1 | 0 | 2 | 1 | 0 | 2 | 0 | 1 | 1 | 0 | 2 | 0 | 0 | 0 | 1 | 0 | 2 | 0 | 0 | 1 | 0 | 1 | 0 | 0 |
| *Cecidostiba geganius* | 1 | 0 | 0 | * | * | 0 | 0 | 0 | 0 | 0 | 1 | 0 | 0 | 1 | 0 | * | 0 | 1 | 0 | 0 | 1 | 0 | 0 | 0 | 0 | 0 | 0 | 0 |
